# The Intersection of Genetic Factors, Aberrant Nutrient Metabolism and Oxidative Stress in the Progression of Cardiometabolic Disease

**DOI:** 10.3390/antiox13010087

**Published:** 2024-01-10

**Authors:** Andrew J. Butcko, Ashley K. Putman, Emilio P. Mottillo

**Affiliations:** 1 Hypertension and Vascular Research Division, Henry Ford Hospital, 6135 Woodward Avenue, Detroit, MI 48202, USA; andrewbutcko@wayne.edu (A.J.B.); putmanas@msu.edu (A.K.P.); 2 Department of Physiology, Wayne State University, 540 E. Canfield Street, Detroit, MI 48202, USA; 3 Department of Large Animal Clinical Sciences, College of Veterinary Medicine, Michigan State University, 784 Wilson Road, East Lansing, MI 48823, USA

**Keywords:** cardiometabolic disease (CMD), metabolic-associated fatty liver disease (MAFLD), non-alcoholic fatty liver disease (NAFLD), chronic kidney disease (CKD) and cardiovascular disease (CVD)

## Abstract

Cardiometabolic disease (CMD), which encompasses metabolic-associated fatty liver disease (MAFLD), chronic kidney disease (CKD) and cardiovascular disease (CVD), has been increasing considerably in the past 50 years. CMD is a complex disease that can be influenced by genetics and environmental factors such as diet. With the increased reliance on processed foods containing saturated fats, fructose and cholesterol, a mechanistic understanding of how these molecules cause metabolic disease is required. A major pathway by which excessive nutrients contribute to CMD is through oxidative stress. In this review, we discuss how oxidative stress can drive CMD and the role of aberrant nutrient metabolism and genetic risk factors and how they potentially interact to promote progression of MAFLD, CVD and CKD. This review will focus on genetic mutations that are known to alter nutrient metabolism. We discuss the major genetic risk factors for MAFLD, which include Patatin-like phospholipase domain-containing protein 3 (*PNPLA3*), Membrane Bound O-Acyltransferase Domain Containing 7 (*MBOAT7*) and Transmembrane 6 Superfamily Member 2 (*TM6SF2*). In addition, mutations that prevent nutrient uptake cause hypercholesterolemia that contributes to CVD. We also discuss the mechanisms by which MAFLD, CKD and CVD are mutually associated with one another. In addition, some of the genetic risk factors which are associated with MAFLD and CVD are also associated with CKD, while some genetic risk factors seem to dissociate one disease from the other. Through a better understanding of the causative effect of genetic mutations in CMD and how aberrant nutrient metabolism intersects with our genetics, novel therapies and precision approaches can be developed for treating CMD.

## 1. Introduction

Globally, the prevalence of obesity has nearly tripled over the past 50 years [1], reaching epidemic levels in much of North America and Western Europe. Current epidemiological data from the Center for Disease Control (CDC) reports that approximately 42% of American adults are obese, putting the US on track to reach the predicted 50% mark by 2030 [2,3]. The persistent rise in weight gain and obesity around the globe has brought about a parallel rise in the prevalence of cardiometabolic disorders, such as metabolic-associated fatty liver disease (MAFLD), chronic kidney disease (CKD) and cardiovascular disease (CVD). With adverse cardiovascular events already being the leading cause of mortality worldwide, the continued rise in obesity prevalence around the globe intensifies concerns surrounding cardiovascular health.

The fundamental cause of weight gain and ultimately obesity is an imbalance between calorie consumption and energy expenditure. Average calorie consumption has increased largely due to a rise in the production of highly processed foods, high in saturated fats, refined carbohydrates and added sugars. Furthermore, calorie expenditure has decreased due to reductions in manual labor and daily physical activity. This imbalance in calorie consumption and expenditure ultimately results in a calorie surplus, which consequently leads to weight gain. Excess calories often take the form of free fatty acids (FFAs), which can be sequestered and stored within adipocytes for later use. The sequestration of excess calories within adipose tissue ultimately causes adipocyte hypertrophy and the tissue to expand. The storage of excess calories in adipocytes is not detrimental per se; however, fat cells have limited ability to store energy as triacylglycerol (TAG). It is the spillover of excess FAs from adipocytes and the ectopic accumulation of fat in key metabolic organs such as the liver, pancreas, kidney and the cardiovascular system that is thought to lead to cardiometabolic disease (CMD) [4,5,6]. Overall, the combination of reduced energy expenditure and increased caloric intake have led to a rise in obesity, lipotoxicity and obesity-related cardiometabolic disorders.

In obesity, enlarged stores of adipose tissue often result in greater FFA release, contributing to a state of dyslipidemia [7]. High levels of plasma lipids are currently the best known indicator of cardiovascular disease risk [8]. This is likely in part due to the vicious positive feedback cycle that occurs with high levels of plasma FFA promoting the release of more FFA through their inhibitory effect on insulin’s antilipolytic actions [9]. In addition to increasing the release of FFAs, expanding adipocytes can release a range of proinflammatory cytokines, such as tumor necrosis factor-alpha (TNF-α), interleukin-6 (IL-6), interleukin 1β (IL-1β) and many others, contributing to a state of low-grade chronic inflammation [10]. These cytokines are known to stimulate the secretion of C-reactive protein, a marker of chronic low-grade inflammation [11]. Interestingly, C-reactive protein levels, much like high levels of plasma lipids, has also been linked to increased risk of coronary artery disease (CAD) [12]. Moreover, C-reactive protein has been suggested to play a role in the pathogenesis of atherosclerosis, the underlying cause of most cardiovascular diseases, though further work is needed to reaffirm this association [13].

Many of the nutrients that drive obesity can induce oxidative stress. Fatty acids (FAs) can cause oxidative stress by protein carbonylation [14] and lipid peroxidation [15]. High glucose can reduce antioxidant defense systems, leading to reactive oxygen species (ROS) production and mitochondrial stress. Fructose can lead to oxidative stress through greater production of superoxide anion [16] and uric acid, which increase ROS production [17]. Additionally, fructose can increase de novo lipogenesis (DNL) and lipid flux from the liver creating greater circulating levels of VLDL and LDL, which promote lipid peroxidation [18].

In addition to dietary factors, genetics also play an important role in determining the risk of MAFLD, CVD and CKD. Genome-wide association study (GWAS) and larger gene exome-wide screens provide information on loci that associate with specific traits. While such studies provide association, mechanistic investigation in cellular and animal models are required to determine the direct cause of such polymorphisms in CMD and their interaction with environmental factors. These polymorphisms can result in loss of function or gain of function mutations, resulting in alterations of metabolic pathways thereby disrupting homeostatic pathways and predisposing to CMD. Importantly, genetic factors and diet can intersect to increase one’s predisposition to disease [19]. While some genetic factors may predispose to certain cardiometabolic disorders, they can also be protective against others. The mechanisms for these contrasting effects on cardiometabolic disorders will be discussed below (see Section 3.3, last paragraph and Section 5.4). While prior reviews covering the topic have summarized and provided insights for how genetics and environmental factors can influence cardiometabolic health [20,21], this review aims to highlight specific gene variants and nutrients that are shown to alter risk of developing CMD, focusing on genetic factors which are known to alter lipid and whole-body metabolism and their underlying mechanisms. Moreover, the role of nutrient excess in driving type 2 diabetes, which also encompasses CMD, has been extensively covered in other reviews and will not be covered [22,23].

In the current review, we examine the interaction of genetics and diet in the progression of MAFLD, CVD and CKD. We first summarize the role of oxidative stress in cardiometabolic disease (Section 2). We then discuss the role of aberrant nutrient metabolism and genetics in MAFLD, CVD and CKD. For MAFLD, we examine the role of fructose (3.1) dietary fatty acids (3.2) and genetics (3.3). For CVD, we examine the role of fatty acids such as saturated and polyunsaturated fatty acids (4.1), simple sugars (4.2) and genetics (4.3). In the section on CKD, we examine the role of ectopic renal fat (5.1), simple sugars and uric acid (5.2), protein-induced kidney damage (5.3) and genes associated with aberrant nutrient metabolism in the progression of CKD (5.4). We also focus on understanding how genetic mutations that cause MAFLD might be cardioprotective and provide insight into the impact of some common genetic mutations that are known to be associated with MAFLD and CVD and how they might impact CKD. Finally, we examine some of the pathways by which MAFLD and CKD intersect, namely epidemiology (6.1), renin-angiotensin system (6.2) and lipid dysregulation (6.3) and conclude with future perspectives (Section 7).

## 2. Oxidative Stress and Cardiometabolic Disease

Oxidative stress refers to an imbalance between the production of free radicals, which include reactive oxygen (ROS) and nitrogen species (RNS; collectively, RONS), and the body’s ability to neutralize them with antioxidants [24]. Examples of RONS include superoxide anion (O_2_^−^), hydrogen peroxide (H_2_O_2_), and nitric oxide (NO) while antioxidants include superoxide dismutase, catalase, peroxidases, and numerous vitamins and minerals (e.g., vitamin E and selenium) [24]. Low concentrations of RONS are necessary for cell signaling and homeostasis [25]. However, when present in excess, RONS contribute to disease pathogenesis [25]. Indeed, oxidative stress is implicated as a major underlying component of several disease pathophysiologies, including metabolic syndrome, type 2 diabetes, atherosclerosis, and MAFLD [26,27,28,29].

Oxidative stress can contribute to CMD pathophysiology via numerous mechanisms, such as endothelial dysfunction, disrupted mitochondrial function, and systemic inflammation [30]. Furthermore, excessive accumulation of RONS can damage DNA, proteins, and lipids, which can then exacerbate the aforementioned mechanisms [25]. For instance, low-density lipoproteins (LDL) oxidized by ROS are readily scavenged by macrophages, resulting in the generation of foam cells that are a major component of atherosclerotic plaques [31,32]. Through its primary receptor, lectin-like oxidized low-density lipoprotein receptor-1, oxidized LDL can then initiate endothelial dysfunction [33]. Endothelial dysfunction is mediated by activated endothelial cells and reduced NO availability [34]. As endothelial cells are activated by inflammatory cytokines, such as those that are induced by ROS, oxidative stress contributes to a dysfunctional endothelium [35]. Additionally, NO reacts with O_2_^−^, forming another reactive molecule called peroxynitrite [34]. Peroxynitrite promotes protein and cell damage, ultimately contributing to cell death [36,37]. Many CMDs are associated with reduced NO, contributing to endothelial dysfunction that then intensifies disease [34].

Mitochondria are one of the major sources of ROS in cells, and therefore can contribute to oxidative stress when their function is disrupted or antioxidant defenses are overwhelmed [38,39]. Importantly, as mitochondria are responsible for FA ꞵ-oxidation, dysfunction can result in excessive circulating FFA and further promote the accumulation of lipids within non-adipose tissues [40,41,42]. Aberrant lipid deposition in non-adipose tissues contributes to CMD and will be discussed in the context of specific diseases below. 

Importantly, excessive nutrients can drive oxidative stress in various tissues, thereby contributing to the development of CMD. For example, high glucose can promote oxidative stress in proximal tubule cells, leading to impaired transport function [43]. Moreover, hypertriglyceridemia can induce ectopic lipid accumulation in key insulin-sensitive tissues and drive oxidative stress [44]. A lipid infusion in healthy patients increases plasma FFA concentration and is sufficient to promote systemic oxidative stress and inflammation [45]. A high fat diet can promote endothelial oxidative stress and inflammation that can progress CVD [46]. Indeed, reversing the accumulation of oxidized phospholipids in the liver can improve MAFLD [47], suggesting interventions that target oxidative stress could be of therapeutic benefit. Furthermore, oxidative stress has been linked to insulin resistance and impaired glucose metabolism exacerbating the development of CMD [48].

## 3. Metabolic-Associated Fatty Liver Disease

Prevalence of non-alcoholic fatty liver disease (NAFLD), recently renamed metabolic-associated fatty liver disease (MAFLD) [49], is on the rise, affecting an estimated 30% of the adult population worldwide [50,51]. It was concluded that the previous nomenclature of “non-alcoholic fatty liver disease” did not reflect current knowledge of the disorder and that “metabolic-associated fatty liver disease” is more informative of disease etiology [52,53]. MAFLD represents a spectrum of disorders, ranging from benign hepatic steatosis (>5% liver fat) to more malignant forms, such as steatohepatitis (>5% liver fat with chronic inflammation and potential fibrosis), liver cirrhosis (>5% liver fat with widespread chronic inflammation and fibrosis), and hepatocellular carcinoma (>5% liver fat with the presence of malignant tumors). MAFLD is most often thought to manifest as a result of sedentary lifestyle and poor dietary eating habits, though genetics factors also play a crucial role in determining patient risk. Lifestyle modifications in diet and exercise have proven sufficient for slowing progression and, in some cases, even reversing MAFLD in the early stages (i.e., hepatic steatosis and steatohepatitis), though not very effective in more advanced fibrotic stages. Some of the benefits of diet and exercise on reducing liver fat may be due to reductions in body mass, as a 10% weight reduction has been shown to improve MAFLD by nearly a full stage in the vast majority of patients [54]. However, dietary changes can also be beneficial independent of weight loss, as Mediterranean style diets high in fish, fruit and olive oil have been shown to reduce liver fat independent of weight reductions, likely due to reductions in refined carbs, saturated fats and added sugars [55]. Moreover, increasing physical activity has been shown to be highly beneficial for reducing liver fat. Exercise, typically defined as planned or scheduled deliberate physical activity, is one of the cornerstones of MAFLD management, as both aerobic and resistance training have proven to yield similar benefits for reducing liver fat [56]. While diet and exercise are largely understood to reduce risk of most all non-hereditary diseases, it is worth noting that changes in physical activity and diet are most often difficult to implement and maintain. Furthermore, some patients are simply unable to make the necessary changes to their physical activity and diet due to a variety of medical, social or economic factors. Importantly, there are still no effective FDA-approved pharmacological treatments for MAFLD, primarily due to an incomplete understanding of the disease pathogenesis. Although GLP-1 agonists which promote weight loss may provide some promise in treating MAFLD, improvements in the resolution of metabolic associated steatohepatitis (MASH) have not been observed [57,58]. Numerous studies have demonstrated a strong association between MAFLD and CVD, though neither a causative relationship nor mechanistic link has yet been proven. However, mounting evidence supports dysregulated metabolism of nutrients, oxidative stress and genetics as major drivers of both conditions.

### 3.1. Fructose and Progression of MAFLD

**Figure 1:**
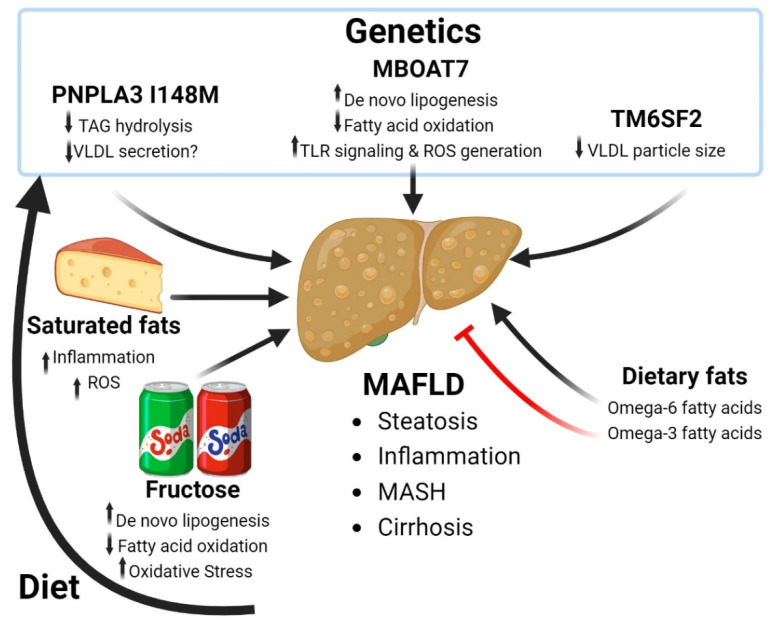
Dietary and genetic drivers in Metabolic Associated Fatty Liver Disease (MAFLD). MAFLD includes a spectrum of liver diseases which can include simple steatosis which can be benign. More advanced stages of disease are marked by inflammation and Metabolic Associated Steatohepatitis (MASH) and cirrhosis of the liver. Fructose can promote de novo lipogenesis in the liver as well as reduce fatty acid oxidation thereby increasing TAG accumulation. Fructose can also have direct effect in promoting oxidative stress in the liver. Saturated fats such as palmitate can also promote TAG accumulation as well as ROS and inflammation in the liver. Dietary fatty acids of Omega-6 and Omega-3 fatty acids can have contrasting effects on MAFLD, although this is area of controversy (see text for details). Genetic factors such as PNPLA3 I148M, MBOAT7 and TM6SF2 can increase the risk for MAFLD. Importantly, diet can interact with genetic factors to further exacerbate the risk for MAFLD.

Fructose is a highly lipogenic substrate for the liver. One of the first studies to show the lipogenic potential of fructose was in diabetic rats where fructose, but not glucose, provided acetate and lactate for de novo synthesis of FAs, demonstrating differential substrate utilization for these sugar sources [59]. Mechanistically, dietary fructose promotes greater lipogenesis through generation of hexose phosphate metabolites which can activate chREBP (Carbohydrate response element binding protein), a master regulator of the DNL transcriptional programs [60,61]. Secondly, the microbial metabolism of fructose generates acetate to feed hepatic DNL, independent of ATP-citrate lyase (ACLY), the rate limiting enzyme for conversion of citrate to acetyl-CoA [62]. These data likely explain a dual mechanism of substrate flux and upregulation of DNL transcriptional programs by which fructose is highly lipogenic. When fed in combination, saturated fat and fructose have been shown to drive a metabolic-associated steatohepatitis or MASH-like phenotype in thermoneutral housed mice, mimicking more closely the pathological condition observed in humans [63]. Interestingly, many of the cardiometabolic symptoms of MASH, liver fibrosis and dyslipidemia can be improved in this model by inducible genetic or pharmacological inhibition of ACLY, [63] demonstrating that targeting the DNL pathway can be of therapeutic benefit. The discrepancy between the two studies may be due to the different dietary models used where liver ACLY deletion occurred from birth and higher concentrations of fructose were administered [62]. Furthermore, rodent and human studies indicate that consumption of fructose reduces fatty acid oxidation through decreased expression of fatty acid oxidation genes (i.e., PPARα) as well as direct effects on modifying the mitochondrial proteome [64,65]. Thus, consumption of fructose has been shown to result in increased synthesis and reduced the breakdown of fats, providing a double hit for lipid accumulation within the liver (Figure 1).

In addition to being a substrate for DNL, fructose has also been shown to directly promote oxidative stress. High fructose intake is associated with increased protein nitration of intestinal tight junction proteins, due to elevated oxidative stress, which results in increased gut leakiness, endotoxemia and steatohepatitis with liver fibrosis, that was partially dependent upon Cytochrome P450 Family 2 Subfamily E Member 1 (CYP2E1) [66]. Fructose consumption has previously been associated with increased fibrosis severity and hepatic inflammation suggesting it might be involved in regulating inflammatory gene expression [67]. 

### 3.2. Dietary Fatty Acids and MAFLD

Dietary FAs are rapidly shuttled to the liver, where they can undergo re-esterification back into TAGs for either storage within lipid droplets or secretion as VLDL cholesterol. Alternatively, once in the liver, FAs can also be shuttled into the mitochondria to be used as a substrate for β-oxidation and the production of adenosine triphosphate (ATP). Excess FA uptake can overwhelm the capacity of the mitochondrial β-oxidation, leading to the uncoupling of mitochondrial respiration and the generation of ROS, a major mediator of low-grade inflammation and progression of MAFLD [68,69] (Figure 1). 

While there have been mixed reports regarding the role of dietary FA saturation on MAFLD, the majority of studies report that a high intake of saturated FAs increases hepatic lipids, while a high intake of unsaturated FAs (i.e., poly- or mono-unsaturated) is associated with reduced hepatic steatosis. With that said, the total calories consumed still appears to be the most important factor when considering diet and risk of MAFLD, as a hypocaloric diet low in fat composition has the same beneficial effect on reducing liver fat as the inverse low-carb hypocaloric diet [70]. However, when total calories consumed are accounted for, simply increasing the percentage of calories from saturated fats results in increased hepatic fat content [71]. On the other hand, consuming a diet rich in mono-unsaturated fatty acids (MUFAs) was shown to reduce liver fat by ~30% in type 2 diabetics [72]. In a randomized double-blind clinical trial, patients receiving MUFAs in the form of olive oil supplementation also displayed a marked reduction in liver fat content post-intervention [73]. Similarly, there is interest in omega-3 (n-3) polyunsaturated fatty acids (PUFAs) for their potential anti-inflammatory properties. The pathways by which n-3 PUFAs reduce oxidative stress and protect against MAFLD have been detailed previously [74]. Briefly, n-3 PUFAs have been shown to be ligands for G-protein coupled receptor 120 (GPR120) where they mediate anti-inflammatory effects and improve insulin sensitivity [75,76]. Moreover, n-3 PUFAs are precursors for an important class of bioactive anti-inflammatory and pro-resolving lipid mediators [77,78]. However, by large, the majority of studies investigating the therapeutic potential of n-3 PUFAs in the treatment of MAFLD report a reduction in liver fat as well as other markers of MAFLD [79]. Though currently the usage of dietary n-3 PUFAs for treatment of MAFLD remains controversial, as not all investigations have been able to reproduce the proposed beneficial effects on reducing liver steatosis [80,81]. In contrast to the effects of n-3 FAs, omega-6 (n-6) FAs have been proposed to be precursors for inflammatory lipid species (i.e., Arachidonic Acid) [82] and promote the progression of MAFLD [83]. However, patients receiving an n-6 PUFA-enriched diet displayed down regulation of Proprotein convertase subtilisin/kexin type 9 (PCSK9), which the authors speculated could be a potential mechanism behind the cholesterol-lowering effects of n-6 PUFAs [84]. Overall, saturated fats are involved in the progression of MAFLD, while the exact role of n-3 and n-6 FAs requires additional mechanistic investigation.

In general, patients with MAFLD seem to consume a diet high in saturated FAs and cholesterol with lower consumption of PUFAs and antioxidants [85,86]. There have been mixed reports regarding the effects of saturated fat content on liver steatosis. Two studies which investigated the effects of saturated fat content on liver fat report that 4 weeks of a diet high in saturated fat has no effect on increasing intrahepatic TAGs in older or overweight patients; however, 4 weeks of a low saturated fat diet reduced intrahepatic TAG levels [87,88]. In contrast, 4 weeks of a hypercaloric diet high in saturated fats (2923 kcal; ~52% sat. fat) resulted in a nearly doubling of intrahepatic TAGs when compared to those fed a calorically controlled diet (2248; ~34% sat. fat) [89]. Moreover, in another hypercaloric model, overfeeding with saturated fat increased intrahepatic TAGs by ~55%, considerably more than overfeeding with unsaturated fat or carbohydrate (5% and 33% increase in hepatic TAGs, respectively), differences that were largely attributed to changes in adipose tissue gene expression with greater upregulation of inflammatory genes in the high saturated fat group [90]. Further evidence supporting that saturated fat is more metabolically harmful for the liver found that rodents fed a diet high in saturated fats had greater hepatic steatosis than did those fed a diet high in fructose [91]. However, when fed in combination, the addition of fructose to a high-fat diet greatly exacerbated hepatic fat accumulation, suggesting an additive effect of fructose on hepatic lipid dysregulation [91]. In conclusion, reducing dietary saturated fat content while increasing intake of unsaturated fats appears to be beneficial for reducing intrahepatic lipids, though a thorough mechanistic understanding is still lacking. 

### 3.3. Genetics of MAFLD

While environmental factors, such as physical activity and dietary factors, play a major role in determining risk of disease, genetics also play a pivotal role in determining susceptibility to CMD. Numerous studies have indicated that genetic variants can significantly influence an individual’s risk of developing these conditions. Understanding the molecular and metabolic pathways that these genetic factors alter will be crucial in elucidating potential molecular targets for future therapeutics. Several genes have been identified that influence the risk of MAFLD, including Patatin-like phospholipase domain-containing protein 3 (*PNPLA3*), Transmembrane 6 superfamily 2 (*TM6SF2*), and Membrane Bound O-Acyltransferase Domain Containing 7 (*MBOAT7*). The variants in these genes have been linked to increased hepatosteatosis and progression of MAFLD (Figure 1).

A common genetic variant in the *PNPLA3* gene (rs738409, I148M), first identified in 2008, is widely known as the greatest genetic determinant of fatty liver disease (mean allele frequency, MAF = 0.2622) [92,93,94]. The association of PNPLA3 I148M with MAFLD was independently confirmed a few years later [95] and in numerous studies which have gone on to link the variant to important liver disease phenotypes, including elevated serum transaminases [92,96], liver fibrosis [96,97,98], and hepatocellular carcinoma [99]. The exact mechanism by which PNPLA3 I148M results in MAFLD is somewhat controversial. The wild-type enzyme exhibits hydrolase [93,100,101] transacylase [102] and acyltransferase activity [103]. Initial studies suggested a gain of function for the variant in acyltransferase activity leading to increased lipid synthesis [103], although this could not be replicated in subsequent studies [100,104]. Neither whole body deletion nor overexpression of wildtype PNPLA3 in mice results in hepatic steatosis [105,106], suggesting that PNPLA3 I148M is not a simple loss of function, but rather a gain of function mutation [100,107]. Genetic knockin of the I148M mutation in mice results in hepatic steatosis, which is greatly exacerbated upon feeding a high sucrose diet [108], supporting a role for the mutant as a potential neomorph [109] that gains a new function. Moreover, knockdown of PNPLA3 in the rat reduces hepatic TAG levels suggesting the WT enzyme has some activity as an acyltransferase to promote fatty acid esterification [110]. Thus, the consensus by which the PNPLA3 variant causes steatosis seems to suggest a mechanism of disruption in hepatic TAG hydrolysis [93,108]. Some studies have suggested that the mechanism by which the I148M variant causes MAFLD is through a loss of lipase function [111,112]. However, this does not explain the fact that deletion of PNPLA3 in mice does not cause hepatic steatosis [105,106] and that expression of PNPLA3 I148M is sufficient to promote TAG accumulation [107]. Moreso, in a chimeric mouse model with engrafted human hepatocytes, PNPLA3 I148M further increased hepatic steatosis in Western diet-challenged mice, providing further evidence for PNPLA3 I148M as a neomorph in the retention of hepatic TAGs [109,113]. More recently, our work demonstrated that PNPLA3 is a novel binding partner of α/β hydrolase domain-containing protein 5 (ABHD5, also known as Comparative gene identification 58; CGI-58), a lipase co-activator enzyme of Patatin-like phospholipase domain-containing protein (PNPLA2) (also known as adipose triglyceride lipase; ATGL), the rate limiting TAG hydrolase in the liver. The PNPLA3 I148M mutation was a gain of function for the interaction with ABHD5, functioning to sequester PNPLA2 away from ABHD5 through a competitive interaction with the co-activator [104]. The interaction of ABHD5 with PNPLA3 was independently confirmed [114]. Thus, it is thought that the reduction in TAG hydrolysis through loss-of-function mechanisms as well as sequestration of ABHD5 function within the liver may lead to a decrease in hepatic production and secretion of very low density lipoprotein particles. 

*TM6SF2* is another gene that when mutated is highly associated with the risk of developing MAFLD. TM6SF2 is abundantly expressed in the small intestine, liver and kidneys of both mice and humans. However, the rs58542926 variant in *TM6SF2*, greatly reduces its expression [115,116] and appears to result in a loss of function as the phenotype observed in KO mice and hepatocyte cell lines mimic that of human patients [116,117]. The *TM6SF2* gene encodes a protein harboring a predicted nine transmembrane domains [116]. Currently the enzymatic function of TM6SF2 is not well understood, though it is believed to have a role in cholesterol metabolism via the mobilization of neutral lipids and lipidation of VLDL particles. The TM6SF2 KO mice were shown to have smaller sized VLDL particles, though the number of newly secreted APOB100, a surrogate marker for the number of VLDL particles, in plasma remained unchanged [118]. Similarly, in vitro experiments in HepG2 and Huh7 cell lines demonstrate that silencing of TM6SF2 results in elevated intracellular TAG content while overexpression resulted in a reduction in intracellular TAGs [117]. These findings suggest that the variant in TM6SF2 (rs58542926, E167K) promotes hepatic steatosis by way of reducing hepatic TAG mobilization and the bulk transfer of neutral lipids into VLDL particles, thereby resulting in hypocholesterolemia and hepatic retention of neutral lipids [119]. Therefore, in patients with the rs58542926 variant, a diet high in fat content is likely to be retained within the liver, further promoting the development and progression of MAFLD.

MBOAT7 is a ubiquitously expressed lysophosphatidylinositol acyltransferase 1 that facilitates the esterification of arachidonoyl-CoA to lysophosphatidylinositol, generating the major molecular species within cell membranes: phosphatidylinositol. The enzymatic activity of MBOAT7, makes it a distinctive contributor to the Land’s Cycle, which through a series of deacylation and reacylation reactions alters phospholipid FA composition, important for generating membrane diversity [120,121]. Alteration of FA saturation within phosphatidylinositols is known to influence the rate of DNL [122,123]. Moreover, the major substrate of MBOAT7, lysophosphatidylinositol, has been proposed to be a crucial mediator for progression of obesity-linked liver disease, as MBOAT7 knockdown in mice treated with lysophosphatidylinositol lipids worsened hepatic inflammatory and fibrotic gene expression [124]. Evidence indicates that the rs641738 mutation promotes development of fatty liver by abolishing MBOAT7’s enzymatic activity, as liver specific genetic knockdown in mice causes spontaneous steatosis within the liver, similar to human patients which express the rs641738 variant [122,125]. Furthermore, MBOAT7 overexpression resulted in mild improvements in hepatic steatosis and markers of liver injury, but garnered no significant improvements in MAFLD pathology overall. However, the lack of improvements in MAFLD pathology could be due to insufficient arachidonoyl-CoA, which could not be ruled out [126]. Mechanistically, the variant has been proven to reduce fatty acid oxidation and increase de novo lipogenesis within the liver via activation of sterol regulatory element binding protein-1 (SREBP1) [122,124,125,127]. Of further interest, MBOAT7 was recently identified as a novel regulator of Toll-like receptor (TLR) signaling [128], and TLR stimulation is known to alter macrophage lipid homeostasis, which in turn promotes the generation of mitochondrial reactive oxygen species [129,130], thereby representing a novel mechanism for how MBOAT7 drives progression of MAFLD.

Recently, a large GWAS meta-analysis using liver imaging and diagnostic-code assessed NAFLD identified 17 genetic loci associated with MAFLD. The above-mentioned loci of *PNPLA3, MBOAT7* and *TM6SF2* were identified in addition to novel loci, such as *Torsin1B* (TOR1B) and *PNPLA2* (*ATGL*) [131]. The mechanism by which PNPLA3 I148M promotes MAFLD is thought to be partly due to reductions in PNPLA2 activity [104,114], while Torsins are nuclear membrane protein/ER resident proteins that function as ATPases, and have previously shown to be involved in the initial lipidation of VLDL particles [120], possibly explaining the GWAS associations with MAFLD. 

Interestingly, adiposity augments the effects of PNPLA3 I148M and TM6SF2 E167K mutations on fatty liver disease without affecting other adiposity-related parameters, suggesting a diet–gene interaction [132]. Indeed, in our own work, the interaction between PNPLA3 I148M and ABHD5 was shown to be augmented by FAs [104], suggesting a mechanism by which diet can interact with this genetic mutation. Moreover, FAs can increase PNPLA3 protein expression by preventing its degradation and carbohydrates increase the transcriptional regulation of PNPLA3 through activation of SREBP1 [133]. With regard to omega FAs and their interaction with gene variants, arachidonic acid (n-6 PUFA) intake has been shown to be associated with increased liver fibrosis in carriers of the PNPLA3 I148M variant [134]. Moreover, in a small randomized control trial, a low n-6:n-3 PUFA ratio was shown to reduce hepatic fat fraction, an effect which was greater in I148M variant carriers [135]. These data suggest that dietary PUFA modulation may be a promising therapeutic treatment for I148M carriers; however, more randomized control trials and mechanistic studies are required to understand how PUFAs affect PNPLA3 I148M driven MAFLD. Overall, these studies suggest that excess consumption of dietary sugars and FAs can further exacerbate the negative effects of fatty liver promoting variants. A summary of the in vitro, in vivo and clinical finding on MAFLD are summarized in Table 1.

While MAFLD is typically associated with and thought to be involved in the etiology of CVD, some genetic mutations that cause MAFLD are protective against coronary artery disease and CVD. The mechanisms by which variants in PNPLA3 and TM6SF2 promote FLD through potential reductions in VLDL secretion are thought to protect carriers from adverse cardiovascular complications through a reduction in plasma lipids [116,117,118,136,137]. However, several studies report no effect of the MBOAT7 mutation on cardiovascular outcomes [138,139] however, others report an association with increased plasma lipids in carriers of the variant as well as greater risk of venous thrombosis. [140,141]. 

## 4. Cardiovascular Disease

Cardiovascular disease is an umbrella term encompassing any diseases of the heart and/or blood vessels. The underlying cause of most cardiovascular diseases and mortality is atherosclerosis, a condition which is characterized by the accumulation of lipids in the intimal layer of the arterial wall. Atherosclerotic plaques most often develop at blood vessel bifurcations and other sites of disturbed laminar flow which in turn experience greater shear stress. Atherosclerosis commonly develops as a result of high levels of blood lipids (i.e., dyslipidemia), in which LDL cholesterol passes through leaky gap junctions in the endothelium and enters the subendothelial space. The LDL particles which are deposited into the subendothelial layer can be oxidized by ROS into oxidized-LDL. The retention of ox-LDL in the subendothelial layer leads to expression of adhesion molecules such as Vascular cell adhesion molecule 1 (VCAM-1), which allow for the recruitment of monocytes. Once recruited, the monocytes can differentiate into macrophages, which recognize and engulf the ox-LDL molecules and generate foam cells. Vascular smooth muscle cells are then recruited to the subendothelial space to produce collagen and elastin forming a fibrous cap around the foam cells and generating the formation of an atherosclerotic plaque [142].

### 4.1. Fatty Acids and Risk of Cardiovascular Disease

**Figure 2:**
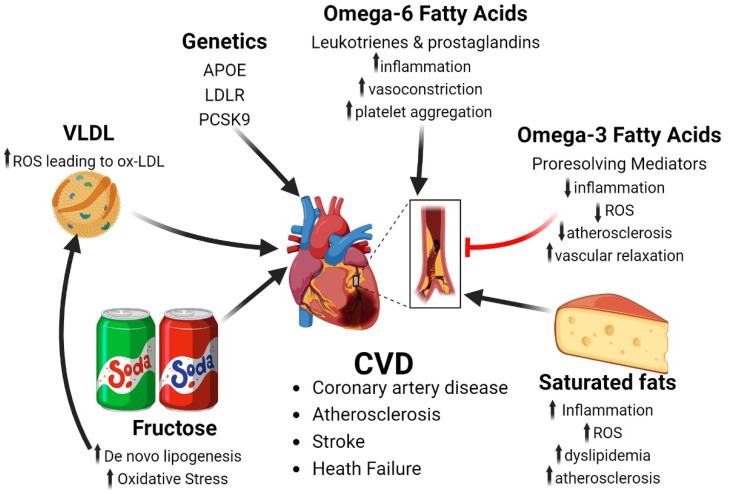
Dietary and other drivers in Cardiovascular Disease (CVD). CVD includes a spectrum disease which can include coronary artery disease, atherosclerosis, stroke and heart failure. Fructose can have direct effect on CVD by increasing oxidative stress. Fructose can also promote de novo lipogenesis in the liver to increase VLDL secretion and delivery of TAGs and cholesterol and the accumulation of ox-LDL in the vascular system. Saturated fats can have direct effect on CVD by increasing inflammation and ROS thereby promoting dyslipidemia and atherosclerosis. Omega-6 fatty acids can act as precursors for proinflammatory lipids leukotrienes and prostaglandins, which can increase inflammation and ROS and promote vasoconstriction and platelet aggregation. Omega-3 fatty acids are precursors for the proresolving lipid mediators that can have protective effects on the vascular system by promoting the resolution of inflammation, decreasing atherosclerosis progression and increasing vascular relaxation.

It has been well documented that high intakes of saturated fatty acids (SFA) negatively impact cardiovascular health through several metabolic pathways, including the promotion of dyslipidemia, atherosclerosis and inflammation [143] (Figure 2). Thus, the American Heart Association along with the World Health Organization recommend for healthy adults to consume a diet that provides <10% of calories from SFA. However, some controversy surrounds the topic of FA modulation for prevention of cardiovascular disease. 

Several recent meta-analyses give mixed reports on the effect of reducing intake of saturated FAs, with some finding no beneficial effects in reducing cardiovascular risk [144,145], others report improvements in cardiovascular outcomes [146,147]. It is possible that much of the variation in results found in the meta-analyses mentioned above could be due to heterogeneity in the exclusion criteria of participants as well as duration of the dietary interventions used within the studies being reviewed. Thus, the role that dietary saturated fats have on adverse cardiovascular outcomes is still unclear; however, the majority of evidence points to improved cardiovascular outcomes for those that reduce intake of saturated fats. This is further supported by work in rodent models which demonstrates that diets high in saturated fats induce systemic inflammation via the release of pro-inflammatory cytokines TNFα, IL-1 and IL-6, in a response that appears to be driven by microbiota release of endotoxin [148]. Furthermore, high dietary consumption of saturated fats has also been shown to induce hyperlipidemia via increasing the expression of Peroxisome proliferator-activated receptor-gamma coactivator 1β (PGC-1β) and SREBP1 in the liver, leading to increased hepatic secretion of lipoprotein particles [149]. While hyperlipidemia is widely recognized as the main driver of atherosclerosis, further evidence from animal studies show that a diet high in saturated fatty acids promotes atherosclerotic plaque buildup [150].

A retrospective re-analysis of the Sydney Diet Heart Study and the Minnesota Coronary Survey suggest that high PUFA intake may increase risk of coronary heart disease mortality [151,152]. Conversely, data from the Oslo Diet–Heart Study show that increasing PUFA intake provides modest protection against recurrent myocardial infarction, angina or sudden death in patients with pre-existing coronary heart disease [153]. Moreover, a recent meta-analysis found that a 5% increase in PUFA intake was associated with an 9% lower multivariate-adjusted risk of heart disease mortality, in those without a prior diagnosis of myocardial infarction but not in patients with heart disease [154]. While several dated randomized control trials demonstrate that replacing intake of SFAs with PUFAs significantly reduces the risk of developing cardiovascular disease and mortality [153,155,156,157,158]. Mechanistically, a meta-analysis of 16-human randomized control trials concluded that consumption of n-3 PUFA is associated with improvements in endothelial function including flow mediated dilation [159]. Moreover, several studies have shown that n-3 FA exhibit anti-inflammatory properties which likely contribute to their cardioprotective effect through reducing systemic inflammation [160,161].

Humans require two essential FAs in their diets, as neither n-3 nor n-6 essential FAs can be synthesized by mammals. It has been noted that the ratio of n-6 to n-3 essential FAs plays an important role in the proposed benefits of PUFA supplementation and reducing risk of cardiovascular disease [162]. An increase in the omega-6/omega-3 fatty acid ratio, in favor of omega-6 PUFAs is prothrombotic and proinflammatory, increasing risk for atherosclerosis, obesity and diabetes [162]. Several investigations into dietary fat composition and LDL oxidation have determined that diets enriched in n-6 FAs lead to greater n-6 FA incorporation into LDL, which promotes the susceptibility of LDL particles to oxidation, thereby promoting the formation of atherosclerotic plaques and coronary artery disease [163,164,165,166]. Additionally, while n-6 FAs (linoleic acid) are metabolized into pro-inflammatory lipid species (i.e., arachidonic acid), n-3 FAs (α-linoleic acid) are metabolized into anti-inflammatory mediators EPA and DHA (20:5 and 22:6, respectively). Arachidonic acid can be further metabolized by cyclooxygenase or lipoxygenase enzymes into prostaglandins and leukotrienes, both critical mediators of a pro-inflammatory response (Figure 2). Both n-3 and n-6 FAs compete with one another for interaction with the same set of metabolizing desaturation, elongation and oxygenase enzymes. Moreover, the lipid mediators that result from the metabolism of n-3 and n-6 FAs serve opposing functions in inflammation, vasoregulation, and platelet aggregation [167]. A class of metabolites derived from n-3 FA metabolism, referred to as specialized pro-resolving mediators (resolvins and maresins), are inflammation-resolving lipids, which have been shown to improve vascular relaxation, reduce arterial inflammation and promote atherosclerotic plaque stability [168,169,170] (Figure 2). Interestingly, DHA has been shown to inhibit NF-kβ activation of cytokine-stimulated ROS production as well as attenuate endothelial cyclooxygenase-2 induction through NADP(H) oxidase and protein kinase Cε (PKCε) inhibition, [171] both of which are thought to be a key mechanism for DHA’s beneficial effects on endothelial function and cardiovascular health. However, when n-6 FAs out-compete n-3 FAs for interaction with their shared enzymes, it leads to the generation of primarily pro-inflammatory mediators (leukotrienes and prostaglandins) as opposed to the anti-inflammatory products of n-3 metabolism (DHA and EPA) [172,173]. In contrast, evidence suggests that diets very high in n-6 FA content may be atherogenic and potentially serve as a substrate to fuel lipid peroxidation and the generation of free-radicals [174]. Similarly, another study which identified individuals that were genetically predicted to have elevated plasma lipid arachidonic acid were positively correlated with incidence of atherosclerosis [175]. Though, other lines of evidence support a cardioprotective role of n-6 FAs [176]. Thus, more work is needed to decipher the role of dietary n-6 FAs on cardiovascular health, specifically those that further investigate the effects of n-6 supplementation on levels of chronic cardiac inflammation, pro-resolving mediator levels, whole-body insulin resistance and plasma lipids as these are potential mechanistic pathways for how n-6 FAs may impact cardiovascular health.

### 4.2. Simple Sugars and Cardiovascular Disease

Current evidence suggests that high consumption of fructose (>30% kcals/day) contributes to risk of CVD, through a variety of mechanisms including a gain in body mass, dyslipidemia and endothelial dysfunction [177]. A meta-analysis of more than 300,000 individuals, investigators found that those with the highest intake of fructose, through sugar sweetened beverages (most often 1–2 drinks/day), were at ~26% increased risk of CVD [178]. As stated above, fructose is a lipogenic substrate for the liver and many of the negative cardiometabolic effects of fructose are likely due in part by the increased lipid flux from the liver through greater secretion of TAGs within VLDL and LDL cholesterol (Figure 2). Both dietary sucrose and fructose increase hyperlipidemia in baboons [179]. Moreover, fructose restriction in obese children with metabolic syndrome improved lipid profiles and insulin sensitivity [180]. Mechanistically, fructose has been shown to induce advanced glycation end-products in rabbits fed a diet high in cholesterol [181]. Moreover, ceramides have been suggested to be intermediary signaling molecules that drive insulin resistance by promoting lipid uptake and impairing glucose utilization [182] and dietary fructose restriction in obese children was shown to reduce ceramide levels and improve insulin sensitivity index over nine days [183].

Several other mechanisms, ranging from inflammation to autonomic overactivity have linked fructose consumption to cardiovascular dysfunction (Figure 2). A diet high in fructose is known to induce cardiac fibrosis and hypertrophy, likely due in part to fructose inhibiting nod-like receptor family pyrin domain containing 4 (NLRP4) a potential negative regulator of pro-inflammatory cytokine secretion [184]. On the other hand, fructose stimulates cardiac inflammation via the recruitment of macrophages to cardiomyocytes, resulting in cardiac remodeling and dysfunction [185]. In terms of sympathetic nervous system activity, a diet high in fructose elevated autonomic outflow to the heart and vasculature, which preceded any alterations in arterial pressure or blood lipids [186]. Finally, a diet high in fructose in mice increased the expression of VCAM-1 independent of plasma cholesterol, suggesting that fructose may cause an increase in expression of vascular adhesion molecules, which may play a role in the generation of atherosclerosis and CVD [187].

In addition to fructose, significant evidence suggests that glycolytic flux is also important in determining the risk of CVD. TP53-inducible glycolysis and apoptosis regulator (TIGAR), a fructose-2,6-bisphosphatase, inhibits glycolysis and directs cellular glucose to the pentose phosphate pathway (PPP). Consequently, diverting carbon sources to PPP results in the production of NADPH which can function as an antioxidant [188]. Thereby, TIGAR is protective against atherosclerosis by limiting ROS and promoting cholesterol efflux from macrophages, suggesting that redirecting glucose away from glycolysis may be beneficial for cardiovascular health [175].

### 4.3. Genetic Risk Factor of Cardiovascular Disease

Several collaborative large scale GWAS have successfully identified numerous genes that are significantly associated with occurrence of CVD [189] (Figure 2). While it is beyond the scope of this review to discuss all of them, it is important to note that genetic predisposition to CVD often requires secondary insult from environmental factors. For instance, single nucleotide polymorphism (SNPs) in apolipoprotein E (APOE) can cause hypercholesterolemia with strong associations for development of coronary artery disease [189]. APOE, a ligand for remnant lipoproteins that functions in the clearance of pro-atherogenic particles, is defective in patients with hypercholesterolemia, resulting in elevated triglyceride-rich remnant lipoproteins in the blood, which promotes development of atherosclerosis and cardiovascular disease [190]. However, disease outcome can be determined by environmental factors such as sedentary lifestyle, high alcohol intake and/or poor dieting in addition to genetic risk [191]. APOE bind the low-density lipoprotein receptor (LDLr) which mediates the uptake of cholesterol from lipoproteins in circulation to the liver, a crucial process in lipoprotein metabolism. Mutations in LDLR can vary in the extent to which they affect post-translational modification, though they all inevitably result in familial hypercholesterolemia and increased risk of developing atherosclerotic cardiovascular disease [192]. In addition, PCSK9 is another gene related to familial hypercholesterolemia and cardiovascular health that is attracting growing attention as a potential target to treat patients at high risk of CVD. PCSK9 impedes hepatic uptake of LDL cholesterol by targeting the LDLr for internalization and degradation, thereby reducing LDL cholesterol lysosomal degradation [193]. Gain of function mutations in PCSK9 results in reduced LDLr levels and subsequently hypercholesterolemia [194], while loss of function mutations increase LDLr levels, thereby lowering circulating LDL cholesterol and providing protection from coronary artery disease. Thus, PCSK9 antibodies (alirocumab and evolocumab) as well as small interfering mRNAs that inhibit intracellular synthesis of PCSK9 (inclisiran) are FDA approved drugs for adults with hypercholesterolemia and established or high risk of CVD [195]. Overall, these studies support the causal link for hypercholesterolemia in disease pathology of CVD. A summary of the in vitro, in vivo and clinical findings on CVD are summarized in Table 2.

## 5. Chronic Kidney Disease

Diabetes and hypertension are the leading causes of CKD, which is defined as decreased glomerular filtration rate (GFR; less than 60 mL/min per 1.73 m^2^), presence of kidney damage biomarkers, or both, for at least 3 months duration [196]. In turn, CKD can exacerbate CMD [197]. While it is widely accepted that these conditions are closely interrelated, the exact mechanisms and links between them remain unclear. As with other CMD, however, increasing evidence supports dysregulated nutrient metabolism, oxidative stress, and genetics as underlying factors influencing CKD [198,199].

### 5.1. Ectopic Renal Fat Accumulation

**Figure 3:**
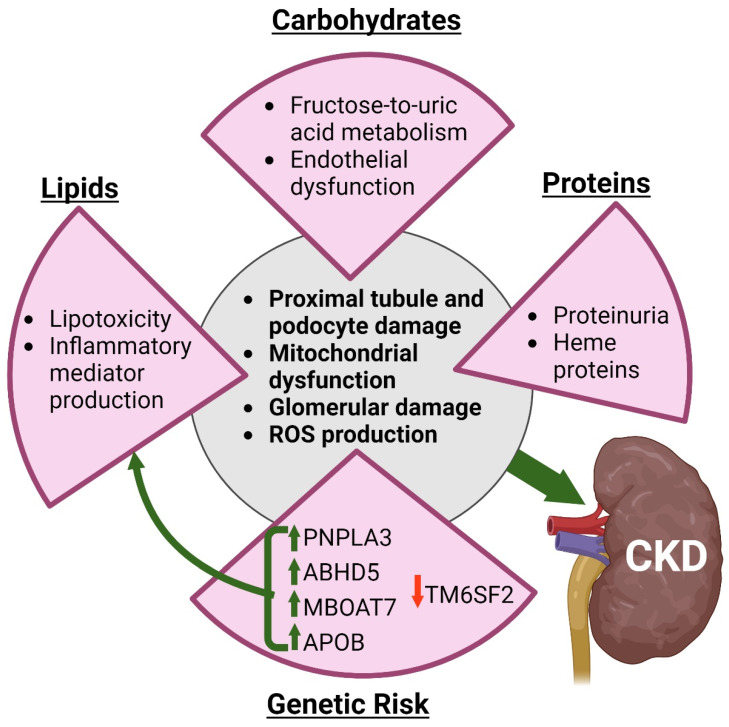
Nutrients and genetic factors contribute to chronic kidney disease (CKD). CKD is marked by proximal tubule and podocyte and glomerular damage and can also be associated with mitochondrial dysfunction and driven by ROS production. Lipids in the form of TAGs can accumulate to promote lipotoxicity in the kidney which can lead to increased inflammatory mediator production. The exact mechanistic pathways by which ectopic lipid accumulation causes CKD are not currently understood but may involve ROS and mitochondrial dysfunction. Carbohydrates such as fructose can be metabolized to uric acid, which can cause oxidative stress and mitochondrial dysfunction in the kidney. Proteins that are not filtered properly can cause proteinuria, which can lead to greater decline in renal function. Moreover, accumulation of heme proteins in the kidney can cause greater injury. Genetic risk factor that affect lipid metabolism can also increase the risk of CKD (PNPLA3 I148M, ABHD5, MBOAT7, APOE) while other seems to be protective (TM6SF2), although the tissue and kidney specific mechanism are not yet understood. Finally, genetic risk factors can interact with lipid pathways to further drive CKD.

Although associations between dysregulated lipid metabolism and CMD have been well-described, the specific links between fatty kidney, CKD, and CMD remain poorly characterized. For instance, do accumulated lipids directly cause cellular damage, or are indirect pathways (e.g., oxidative stress and inflammation) activated by lipids to promote disease? In any case, increasing evidence suggests that ectopic renal fat accumulation contributes to CKD [200] (Figure 3). 

For decades, studies have supported direct toxic effects of excessive lipid deposition in non-adipose tissues [201,202]. Indeed, the first reports of nephron lipotoxicity came from Moorhead and colleagues in 1982 [203]. They posited that following an inciting glomerular injury that caused albuminuria, the liver would produce compensatory lipoproteins leading to hyperlipidemia. These changes could then perpetuate glomerular or tubulointerstitial disease [203]. Sustained or progressive damage can lead to CKD. On the other hand, FAs might indirectly influence CKD outcomes via the production of numerous lipid mediators [204]. Arachidonic acid-derived products, such as leukotrienes, can promote kidney damage through leukocyte recruitment [205]. In contrast, 8,9-epoxyeicosatrienoic acid and 20-hydroxyeicosatetraenoic acid have been described as protective for the glomerular filtration barrier [206,207]. Although the exact mechanisms underlying ectopic renal lipid accumulation and CKD remain poorly defined, dyslipidemia has potential to disrupt the kidney’s function as a major regulator of metabolism. 

Intrarenal lipid accumulation is most commonly documented in renal proximal tubule epithelial cells (RPTEC), podocytes, and mesangial cells [208]. Systemic inflammation, such as that commonly encountered with CMD, promotes renal lipid accumulation that can further exacerbate kidney fibrosis [209]. Furthermore, RPTEC constantly reabsorb and secrete solutes to maintain homeostasis, and thus, have intense energy demands [210,211]. Unsurprisingly then, FA oxidation is a critical energy production pathway for RPTEC as it is for other highly metabolic tissues, including the heart [212]. However, too much FA exposure or dysregulated FA oxidation can negatively impact mitochondrial function, as is known to happen in skeletal muscle [213]. While there is substantial support for these types of detrimental effects in non-renal tissues, less is available for the kidney itself. In a study investigating the role of the antioxidant sirtuin 3 in preventing lipotoxicity, palmitic acid (PA) caused increased mitochondrial ROS and decreased oxidative capacity of proximal tubules when sirtuin 3 was knocked out [214]. Although this study investigated lipotoxicity in the context of overexpressing or knocking out a specific antioxidant, one may be able to speculate that the same mitochondrial dysfunction would occur if excess PA was overwhelming the cell. However, without more studies that specifically investigate questions related to how excess FA affect kidney function, it will be difficult to understand its impact in CKD. Indeed, a major limitation to currently available studies regarding the relationship between and mechanisms underlying fatty kidney, CKD, and disrupted renal nutrient metabolism is the lack of available models. To address this constraint, kidney-specific knockout or overexpression models of altered lipid metabolism should be developed, paying particular attention that they are not confounded by other whole-body metabolic alterations, such as impaired glucose handling. An excellent example comes from the work of Onodera et al., who successfully developed tubule-specific adiponectin knockout and overexpression mouse models. In doing so, the authors were able to better understand the importance of renal adiponectin to gluconeogenesis and implicated the accumulation of ceramides in kidney dysfunction [215].

Unlike RPTEC, podocytes rely primarily on glucose utilization as opposed to fatty acid metabolism [216]. Regardless, lipids may exert toxic effects in this cell type as well. In cultured podocytes, PA induced mitochondrial superoxide and hydrogen peroxide formation, which was implicated in the progression of diabetic nephropathy [217]. Xu et al. found that PA induced mitochondrial and cytosolic ROS, ER stress, and apoptosis while altering mitochondrial morphology and metabolism [218]. Furthermore, podocytes became insulin resistant when treated with PA for 24 hr, although the mechanism in which it did so was not elucidated [219]. Although studies demonstrate correlations between PA and dysfunctional podocytes, clear mechanistic evidence remains scarce. Therefore, it will be important to directly interrogate these mechanisms to gain a better understanding of overly abundant fatty acids and CKD.

One possible explanation in how overabundant FA may cause dysfunction is that kidney cells need to enhance metabolism via increased mitochondrial abundance and activity to handle them [47,220]. As mitochondria are the most significant source of ROS in aerobic organisms, increased mitochondrial content or activity can contribute to heightened ROS burden [220,221]. However, decreased β-oxidation can also result in mitochondrial dysfunction that contributes to disease [222]. Indeed, decreased FA oxidation was implicated as the primary driver of fibrosis in a study by Kang et al. They found that kidney samples from humans with decreased GFR and histological evidence of fibrosis had markedly downregulated genes associated with FA oxidation [212]. Moreover, mice were more protected from renal fibrosis when FA oxidation was enhanced via Ppargc1a overexpression or fenofibrate administration [212]. The detrimental effects did not seem to stem from increased lipid content, as overexpression of the long-chain FA transporter, CD36, did not cause increased susceptibility to renal damage compared to control mice, despite differences in fat accumulation. Thus, a sophisticated balance of mitochondrial activity and ROS production is necessary for maintaining physiological processes. 

### 5.2. Simple Sugars and Uric Acid

Besides lipids, simple sugars such as fructose have been implicated in kidney dysfunction. Endogenous fructose production in the kidney is limited to the proximal tubule [223]. In a fructokinase knockout model of diabetic mice (rendering them incapable of producing endogenous fructose), renal damage was reduced and function was improved compared to wild-type diabetic mice, despite similar levels of hyperglycemia [223]. As no fructose was provided in the diet, this suggests that endogenous tubular production of fructose can contribute to diabetic nephropathy [223]. The authors suggested that fructose may cause tubular damage via the production of oxidants and uric acid [223]. Fructose also causes an increase in proinflammatory cytokine production, which can further exacerbate oxidative stress [224,225].

As mentioned above, the metabolism of fructose utilizes ATP in which the generated AMP is metabolized to uric acid. Uric acid affects many physiologic processes that contribute to CKD. For instance, mild hyperuricemia causes proximal tubule dysfunction and further elevations will cause deposition of urate crystals into the kidney [226,227]. Many other proposed mechanisms involve oxidative stress [226]. Indeed, metabolism of xanthine to uric acid results in the formation of H_2_O_2_ and intracellular uric acid itself acts as a prooxidant [228,229]. Uric acid additionally decreases nitric oxide and therefore supports endothelial dysfunction [230]. Thus, fructose and its metabolite uric acid can contribute to oxidative stress that further exacerbates disease.

Similar to increased lipid loading in the kidney which leads to heightened mitochondrial activity, excessive carbohydrates can also cause mitochondrial dysfunction. An example exists in cases of type II diabetes, where hyperglycemia promotes increased mitochondrial activity to metabolize the excess glucose, resulting in enhanced ROS formation. The increased oxidative state can then cause mitochondrial dysfunction [231]. Likewise, uric acid also alters mitochondrial activity [232]. In this case, uric acid uncouples fructose metabolism from mitochondrial respiration. However, other metabolic pathways such as lipid synthesis are still supported [232,233,234]. This is one mechanism in which fructose and uric acid contribute to fatty liver disease, and it could be possible that it contributes to fatty kidney as well [234]. Further investigation into the fructose and uric acid metabolic pathways would be beneficial to better understanding kidney health and disease (Figure 3).

### 5.3. Protein-Induced Kidney Damage

Finally, proteins are another class of nutrients that can contribute to CKD. Glomerular diseases that result in proteinuria lead to declines in renal function [235,236]. This effect largely seems to be mediated through tubule damage. For instance, treating human proximal tubular HK-2 cells with excessive urinary protein led to apoptosis [237]. Primary mouse RPTEC or HK-2 cells treated with albumin demonstrated decreased numbers of autophagosomes, suggesting impaired autophagy [238]. Urine proteins may promote CKD through the activation of the unfolded protein response [239]. Plasma proteins may also promote inflammation in RPTEC, contributing to CKD [240]. Heme proteins additionally contribute to kidney disease via several mechanisms reviewed elsewhere [241,242,243]. As with lipids and simple sugars, ROS production is a significant contributing factor to cellular damage [244]. Hence, protein-induced renal damage is likely mediated through numerous interrelated factors, such as inflammation and oxidative stress (Figure 3). A summary of the influence of nutrient metabolism on CKD is presented in Figure 3 and a summary of the in vitro, in vivo and clinical findings on CKD are summarized in Table 3.

### 5.4. Genes Associated with Aberrant Nutrient Metabolism and CKD

While various SNPs have been associated with CKD and renal end stage failure [245,246,247] we will focus on genetic factors related to nutrient metabolism that influence CKD. Certain genetic mutations of *PNPLA3* have been recently associated with impaired kidney function. Obese children homozygous for the I148M mutation of *PNPLA3* had decreased GFR compared to children with other genotypes and this effect was exacerbated in children with NAFLD [248]. Similarly, Di Sessa and colleagues assessed 1036 pediatric patients, some of which were clinically normal, some were prediabetic, and finally, some carried the I148M polymorphism of *PNPLA3*. Patients with prediabetes had lower GFR compared to those with normal glucose tolerance and this effect was further exaggerated in those with the I148M mutation [249]. Other regulators of lipid homeostasis, such as *ABHD5*, can also result in kidney disturbances if mutated. One case report details a male with a homozygous mutation in *ABHD5*, resulting in Chanarin–Dorfman syndrome, otherwise known as neutral lipid storage disease with ichthyosis [250]. This condition is characterized by congenital ichthyosiform erythroderma and accumulation of neutral lipid vacuoles in leukocytes, liver, eyes, kidneys, and other tissues. Rarely, as in this case, patients with Chanarin–Dorfman syndrome present with proteinuria [250]. Currently, little is known about the mechanisms for how the PNPLA3 I148M might contribute to CKD. One idea is that the variant might lead to an accumulation of lipids within the podocytes leading to a subsequent increase in inflammation [251], as the *PNPLA3* gene has been shown to be highly expressed in kidney podocytes and lipid accumulation in these cells has been linked to obesity-related glomerulopathy [252]. Alternatively, PNPLA3 I148M may disrupt the function of ABHD5 as had been proposed for the liver [104]. A mutation in *MBOAT7*, which is another genetic risk factor for NAFLD, is also associated with greater CKD [253]. The rs626283 variant of *MBOAT7*, which seems to result in a loss of function mutation in phosphatidylinositol metabolism [254] was associated with a greater CKD disease stage [253]. Further large-scale analysis studies are required to determine the association between rs626283 and CKD. 

In contrast, patients with a particular mutation in transmembrane member 6 superfamily 2 (*TM6SF2*) seem to be protected from renal disease. Musso et al. found that nondiabetic, nonobese adults with the TM6SF2 E167K polymorphism had increased GFR with less albuminuria and CKD compared to individuals without the mutation [255]. Later, Marzuillo et al. recapitulated the increased GFR trend in obese children with the mutation. The significant positive correlation was seen in patients with and without NAFLD, although it was stronger in the former group [256]. It is interesting to note that mutations in *PNPLA3*, *MBOAT7*, and *TM6SF2* are associated with fatty liver disease; however, *TM6SF2* has a contrasting effect on CKD compared to mutations in *PNPLA3* and *MBOAT7*. As the links between diet, genes associated with lipid metabolism, and CKD are still poorly understood, further studies will be necessary to understand the mechanisms underlying the effects of these mutations on kidney function and would be benefitted by kidney-specific models of aberrant nutrient metabolism. Nevertheless, the effect of *PNPLA3* mutations are well-understood in the liver so one may be able to speculate that similar interactions (see Section 3.3 above) may occur in the kidney.

GWAS loci that have been associated with CVD, also have been implicated in CKD. Among polymorphisms in PKCS9 and Apolipoprotein B-100 (ApoB), mutations in *ApoB* were identified as being associated with greater risk of developing kidney disease among diabetic patients [257]. Indeed, ApoB levels have been correlated with a decline in eGFR [258]. Moreover, patients with familial hypercholesterolemia have reduced eGFR and are at greater risk of CKD [259,260]. Overall, these studies further support a role for dyslipidemia as a driving factor in CKD; however, further mechanistic studies are required to understand the direct relationship between these two diseases. A more in depth analysis of lipid abnormalities in CKD is provided elsewhere [261]. Conversely, CKD can also exacerbate CVD as a mouse model of kidney impairment has been shown to increase atherosclerotic progression, effects which were dependent on IL-17a [262] Gene mutations and their reported effects on various CMD are reported in Table 4.

## 6. Intersection of MAFLD and CKD

While significant evidence links CVD with MAFLD, with the two typically going hand-in-hand, the links between MAFLD and CKD is less understood. As discussed above, some common genetic mutations that increase risk for MAFLD also increase the risk to CKD, but others seem to dissociate the two from one another. Below we discuss some of the evidence linking MAFLD to CKD.

### 6.1. Epidemiology of MAFLD and CKD

Due to parallels in traditional risk factors and comorbidities, as well as a lack of prospective studies, it has been difficult to determine a causational relationship between MAFLD and CKD [263]. Nevertheless, increasing evidence links the presence of MAFLD with increased incidence of CKD. A meta-analysis of 11 cross-sectional and 9 longitudinal human studies (29,282 participants) shows that the presence of MAFLD is associated with a 2-fold greater risk of CKD development (increased prevalence odds ratio of 2.12), which remained significant after adjusting for the covariates age, BMI, metabolic syndrome, diabetes, smoking status, ethnicity, cirrhosis, waist circumference, (Homeostatic Model Assessment -index, and duration of follow-up [264]. In terms of steatosis and fibrosis, it was strongly correlated with increases in prevalence and severity of CKD [264]. Another meta-analysis study reported that prevalence of CKD increased in individuals with MAFLD, in both diabetic and non-diabetic populations [263]. It was found that among patients with MAFLD incidence of CKD increased to 20–55% compared with 5–35% in those without MAFLD [263].

To date, there is a growing number of prospective longitudinal cohort studies which consistently show that MAFLD, diagnosed by either biopsy, ultrasonography, or liver enzyme levels, is significantly associated with an increased incidence of CKD [263,265,266,267,268,269]. Worth mentioning is that most of these studies retained a significant association between MAFLD and CKD even after controlling for numerous confounding factors. For example, a systematic review of nine observational studies found that over a median follow-up duration of 5.2 years, MAFLD was associated with a nearly 40% increased risk of development for CKD, even after adjusting for common risk factors and potential confounding variables, such as age, sex, BMI, hypertension, smoking, diabetes, baseline GFR, and the use of certain medications [270]. While data from cross-sectional and retrospective studies are robust, analysis of prospective randomized control trials is needed to determine a causal effect of MAFLD on driving CKD.

### 6.2. Renin-Angiotensin System Activation

While there are numerous factors that link the two conditions, it has been suggested that altered renin-angiotensin system (RAS) activation and dysregulation of lipid metabolism leading to impaired antioxidant defense are key points of focus for researchers investigating the association between MAFLD and CKD [271]. Interestingly, adipocytes have been shown to produce up to 30% of the total circulating angiotensinogen as well as other components of the RAS system at lower levels [272]. Moreover, the expression of RAS components within adipocytes seems to be nutritionally regulated, as it has been shown that fasting can produce increases in angiotensinogen mRNA expression within white adipose stores [273]. Similarly, in rodent models, hyperglycemia has been shown to induce angiotensinogen expression in white adipose tissue [274]. Thus, it can be suggested that in states of excess adiposity and metabolic dysregulation, such is commonly seen in MAFLD, constituents of the RAS system may be overexpressed subsequently leading to a state of chronically increased RAS activation.

Within MAFLD, RAS activation is known to promote hepatic fibrosis while RAS blockade using angiotensin II receptor blockers, such as losartan and olmesartan, or angiotensin-converting enzyme inhibitors, such as perindopril and lisinopril, have been shown to mitigate the advancement of fibrosis in patients with MASH [275]. More specifically, angiotensin II is reported to have a detrimental impact on the liver, as it promotes hepatic insulin resistance, DNL and production of IL-6 and tumor growth factor-β proinflammatory cytokines [255]. Whereas, RAS activation in the kidney is implicated in renal ectopic fat deposition, which is a known factor contributing to oxidative stress and inflammation via its impact on glomerular hemodynamics, particularly through its effects on efferent arteriole vasoconstriction [276]. 

### 6.3. Lipid Dysregulation

As previously mentioned, substantial epidemiological evidence suggests that MAFLD is an independent risk factor for CKD. However, there is also research which states that metabolic syndrome is also involved in the progression of CKD, at least in part, through activation of hepatic macrophages. Mechanistically, metabolic syndrome has been said to induce a state of chronically increased levels of FFA in the plasma, mainly due to an inability to suppress FA release from adipocytes by way of insulin insensitivity [277]. This can result in an abundance of FAs which circulate to the liver, leading to hepatic macrophage activation creating a proinflammatory cytokine response, which further perpetuates insulin resistance [278]. Additionally, the activation of hepatic macrophages has been shown to increase activity of the renin-angiotensin-aldosterone system and oxidative stress, thereby promoting vascular and renal damage [279]. Thus, altered nutrient metabolism, inflammation, and oxidative stress likely bridge MAFLD and CKD.

## 7. Conclusions and Future Perspectives

The incidence of CMD, which includes MAFLD, NAFLD and CKD, has reached epidemic proportions due to increased consumption of diets high in saturated fats and fructose and modified lifestyles. Further understanding the mechanisms by which these nutrients mediate their detrimental effects and how they interact with our genes will be of utmost importance to identify the targets of therapeutic interest. In addition, understanding the tissue-specific mechanisms by which some genetic mutations increase susceptibility to certain aspects of CMD but are protective against others will be another area of continued focus. Moreover, GWAS continues to identify risk factors that promote [131] or protect [280,281] from cardiometabolic disorders such as MAFLD. Identifying the mechanisms of these protective variants should provide additional therapeutic targets. The current review is not without its limitations, as many of the studies cited herein were conducted in either isolated cell cultures or animal models and thus further investigations are required for translation of the findings to human health. Moreover, some of the topics covered, such as the effects of n-6 fatty acids on inflammation and cardiovascular health, remain contentious in the field. All sources of information used in the generation of this review were retrieved from PubMed.gov or the Centers for Disease Control and Prevention and the World Health Organization’s public websites. 

## Figures and Tables

**Table 1:** Summary of studies of interest investigating MAFLD.

Model	Subject (Gene/Nutrient)	Major Findings	Citation
In vitro	Fructose	Activates chREBP and DNL	[60]
	PNPLA3 I148M	promotes MAFLD through reducing hepatic TAG hydrolysis; sequestration of ABHD5	[93,100]; [104,114]
	TM6SF2	Involved in secretion of hepatic TAGs	[117]
	MBOAT7	depletion increases hepatic TAGs	[123]
In vivo	Fructose	Provides lactate and acetate for DNL	[59]
		Metabolites activate chREBP	[33]
		Increases lipogenesis independent of ACLY	[62]
	ATP-citrate Lyase (ACLY)	Inhibition reduces liver fat and ballooning; reduces blood glucose, TAGs and cholesterol	[63]
	High fat vs. high fructose diet	Dietary fat and cholesterol are primary drivers of MAFLD	[91]
	PNPLA3	PNPLA3 deficiency does not promote hepatic steatosis; nor does overexpression, I148M is gain of function	[105,106]; [107]
	TM6SF2	TM6SF2 is required for VLDL assembly	[118]
	MBOAT7	Loss of MBOAT7 promotes MAFLD while overexpression improves	[122,124,125,127]; [126]
Clinical	Fructose	High consumption of fructose associates with greater fibrosis	[67]
	Mitochondrial activity	MAFLD reduces mitochondrial activity	[68]
	Fatty Acids	Hypocaloric diet low in fat harbors same benefits as hypocaloric diet low in carbs	[70]
		MUFA enriched diet reduces hepatic steatosis	[72]
		n-3 PUFA supplementation improves MAFLD n-3 PUFA supplementation improves MAFLD	[73,80,81,128] [73,80,81,128]
		HFD increases AA in phospholipid fraction of liver	[83]
		N-6 supplementation reduced liver fat relative to high saturated fat diet	[84]
		Amount of dietary fat influences liver fat content	[71]
		Low fat diet reduced liver TAGs. No effect of HFD	[88]
		Saturated fat is more metabolically harmful for liver	[90]
	Dietary patterns in MAFLD patients	MAFLD patients consume diets rich in saturated fat	[86]
		MAFLD patients consume a diet rich in sat. fat and majority are deficient in PUFAs and MUFAs	[85]
	TM6SF2	rs58542926 promotes MAFLD progression	[115]
	PNPLA3	Association with MAFLD, MASH, cirrhosis	[92,93,94,95,96,97,98,99]
		PNPLA3 I148M affects VLDL secretion	[136]

**Table 2:** Summary of studies investigating CVD.

Model	Subject (Gene/Nutrient)	Major Findings	Citation
In vivo	PUFAs and CVD	PUFAs protect against CAD (non-human primates)	[150]
	Fructose and CVD	Fructose consumption exerts negative effects on CV health	[177,179,180,181,184,185,186,187]
Human studies	Dietary Fat and CAD in women	Saturated and trans fats increase risk of CAD	[143]
Clinical	PUFAs and CVD	Replacement of sat. fat with veg oil reduces risk of CHD	[153,155,156]
		n-3 PUFAs negate adverse LV remodeling after MI	[161]
Meta-analysis	Dietary fat modulation and risk of CVD	No effect observed	[144,145,151]
		Replacing Sat. fat with PUFAs lowers risk of CVD	[146,147,157,158]
		Replace sat. fat with **n-6** PUFA increases CVD death	[152]
	n-3 PUFAs and endothelial function	n-3 supplementation improves endothelial function	[159]
	n-3/n-6 ratio and CVD risk	n-3/n-6 ratio important for CVD risk	[162,167,173,174,176]
	APOE; LDLr; PCSK9	LDL promotes atherosclerosis and CVD	[189]; [192]; [194,195]
GWAS	Lipid metabolism genes	Genetic variants influence risk of CVD	[141,189]

**Table 3:** Summary of studies investigating aberrant nutrient metabolism and CKD.

Model	Subject (Gene/Nutrient)	Major Findings	Citation
In vitro	Lipid-derived mediators	Exaggerates or protects against CKD depending on mediator and context	[204]; [205]; [207]
	Palmitic acid	Increased mitochondrial ROS and decreased oxidative capacity in RPTEC; decreased cytosolic and mitochondrial ROS, ER stress, apoptosis, and insulin resistance in podocytes	[214]; [217]; [218]; [219]
	Albumin	Tubule apoptosis; decreased autophagosome number	[237]; [238]; [244]
	Urinary protein	Increases ROS-mediated activation of ERK, leading to tubule damage and apoptosis	
In vivo	Lipid	Increased renal fibrosis	[209]
	Lipid-derived mediators	Exaggerates or protects against CKD depending on mediator and context	[204]; [205]; [206];
	Fructose	Increased renal damage	[223]
	Uric acid	Exacerbates tubule injury	[227]
Meta-analyses	Albumin	Higher risk of CKD when increased in urine	[235]; [236]

**Table 4:** Gene mutations and their effect on risk of developing MAFLD, CVD and CKD.

Gene Mutation	MAFLD	CVD	CKD	Result of Mutation on Function
PNPLA3 rs738409	Increase	Decrease	Increase	Gain of function /Neomorph
TM6SF2	Increase	Decrease	Decrease	Loss of function
MBOAT7	Increase	No effect	Increase	Loss of function
APOB		Increase	Increase	Loss of function
LDLr	Increase	Increase	Unknown	Loss of function
PCSK9	GOF = increase LOF = no effect	GOF = increase LOF = decrease	Unknown	Both GOF and LOF identified

## References

[1] WHO Obesity and Overweight. https://www.who.int/news-room/fact-sheets/detail/obesity-and-overweight#:~:text=Worldwide%20obesity%20has%20nearly%20tripled,%2C%20and%2013%25%20were%20obese.

[2] Ward Z.J., Bleich S.N., Cradock A.L., Barrett J.L., Giles C.M., Flax C., Long M.W., Gortmaker S.L. (2019). Projected U.S. State-Level Prevalence of Adult Obesity and Severe Obesity. N. Engl. J. Med..

[3] Control C.f.D. The High Obesity Program (HOP 2023). https://www.cdc.gov/nccdphp/dnpao/state-local-programs/fundingopp/2023/hop.html.

[4] Virtue S., Vidal-Puig A. (2010). Adipose tissue expandability, lipotoxicity and the Metabolic Syndrome—An allostatic perspective. Biochim. Biophys. Acta.

[5] Unger R.H., Clark G.O., Scherer P.E., Orci L. (2010). Lipid homeostasis, lipotoxicity and the metabolic syndrome. Biochim. Biophys. Acta.

[6] Britton K.A., Fox C.S. (2011). Ectopic fat depots and cardiovascular disease. Circulation.

[7] Klop B., Elte J.W., Cabezas M.C. (2013). Dyslipidemia in obesity: Mechanisms and potential targets. Nutrients.

[8] Pai J.K. (2004). Inflammatory Markers and the Risk of Coronary Heart Disease in Men and Women. N. Engl. J. Med..

[9] Jensen M.D. (1989). Influence of Body Fat Distribution on Free Fatty Acid Metabolism in Obesity. J. Clin. Investig..

[10] Fain J. (2006). Release of Interleukins and Other Inflammatory Cytokines by Hunman Adipose Tissue is Enhanced in Obesity and Primarily due to the Nonfat Cells. Vitam. Horm..

[11] Calabro P., Willerson J.T., Yeh E.T. (2003). Inflammatory cytokines stimulated C-reactive protein production by human coronary artery smooth muscle cells. Circulation.

[12] Emerging Risk Factors C., Kaptoge S., Di Angelantonio E., Pennells L., Wood A.M., White I.R., Gao P., Walker M., Thompson A., Sarwar N. (2012). C-reactive protein, fibrinogen, and cardiovascular disease prediction. N. Engl. J. Med..

[13] Badimon L., Pena E., Arderiu G., Padro T., Slevin M., Vilahur G., Chiva-Blanch G. (2018). C-Reactive Protein in Atherothrombosis and Angiogenesis. Front. Immunol..

[14] Hauck A.K., Huang Y., Hertzel A.V., Bernlohr D.A. (2019). Adipose oxidative stress and protein carbonylation. J. Biol. Chem..

[15] Murdolo G., Piroddi M., Luchetti F., Tortoioli C., Canonico B., Zerbinati C., Galli F., Iuliano L. (2013). Oxidative stress and lipid peroxidation by-products at the crossroad between adipose organ dysregulation and obesity-linked insulin resistance. Biochimie.

[16] Delbosc S., Paizanis E., Magous R., Araiz C., Dimo T., Cristol J.P., Cros G., Azay J. (2005). Involvement of oxidative stress and NADPH oxidase activation in the development of cardiovascular complications in a model of insulin resistance, the fructose-fed rat. Atherosclerosis.

[17] Albert-Garay J.S., Riesgo-Escovar J.R., Salceda R. (2022). High glucose concentrations induce oxidative stress by inhibiting Nrf2 expression in rat Muller retinal cells in vitro. Sci. Rep..

[18] Moore J.B., Gunn P.J., Fielding B.A. (2014). The role of dietary sugars and de novo lipogenesis in non-alcoholic fatty liver disease. Nutrients.

[19] Heianza Y., Qi L. (2017). Gene-Diet Interaction and Precision Nutrition in Obesity. Int. J. Mol. Sci..

[20] Drozdz D., Alvarez-Pitti J., Wojcik M., Borghi C., Gabbianelli R., Mazur A., Herceg-Cavrak V., Lopez-Valcarcel B.G., Brzezinski M., Lurbe E. (2021). Obesity and Cardiometabolic Risk Factors: From Childhood to Adulthood. Nutrients.

[21] Heianza Y., Qi L. (2019). Impact of Genes and Environment on Obesity and Cardiovascular Disease. Endocrinology.

[22] Marcadenti A. (2016). Diet, Cardiometabolic Factors and Type-2 Diabetes Mellitus: The Role of Genetics. Curr. Diabetes Rev..

[23] Lombardi R., Iuculano F., Pallini G., Fargion S., Fracanzani A.L. (2020). Nutrients, Genetic Factors, and Their Interaction in Non-Alcoholic Fatty Liver Disease and Cardiovascular Disease. Int. J. Mol. Sci..

[24] Lushchak V.I. (2014). Free radicals, reactive oxygen species, oxidative stress and its classification. Chem. Biol. Interact..

[25] Valko M., Leibfritz D., Moncol J., Cronin M.T., Mazur M., Telser J. (2007). Free radicals and antioxidants in normal physiological functions and human disease. Int. J. Biochem. Cell Biol..

[26] Francisqueti F.V., Chiaverini L.C., Santos K.C., Minatel I.O., Ronchi C.B., Ferron A.J., Ferreira A.L., Correa C.R. (2017). The role of oxidative stress on the pathophysiology of metabolic syndrome. Rev Assoc Med Bras.

[27] Rehman K., Akash M.S.H. (2017). Mechanism of Generation of Oxidative Stress and Pathophysiology of Type 2 Diabetes Mellitus: How Are They Interlinked?. J. Cell Biochem..

[28] Forstermann U., Xia N., Li H. (2017). Roles of Vascular Oxidative Stress and Nitric Oxide in the Pathogenesis of Atherosclerosis. Circ. Res..

[29] Videla L.A., Rodrigo R., Orellana M., Fernandez V., Tapia G., Quinones L., Varela N., Contreras J., Lazarte R., Csendes A. (2004). Oxidative stress-related parameters in the liver of non-alcoholic fatty liver disease patients. Clin. Sci..

[30] Swiatkiewicz I., Wroblewski M., Nuszkiewicz J., Sutkowy P., Wroblewska J., Wozniak A. (2023). The Role of Oxidative Stress Enhanced by Adiposity in Cardiometabolic Diseases. Int. J. Mol. Sci..

[31] Palinski W., Rosenfeld M.E., Yla-Herttuala S., Gurtner G.C., Socher S.S., Butler S.W., Parthasarathy S., Carew T.E., Steinberg D., Witztum J.L. (1989). Low density lipoprotein undergoes oxidative modification in vivo. Proc. Natl. Acad. Sci. USA.

[32] Haberland M.E., Mottino G., Le M., Frank J.S. (2001). Sequestration of aggregated LDL by macrophages studied with freeze-etch electron microscopy. J. Lipid Res..

[33] Pirillo A., Norata G.D., Catapano A.L. (2013). LOX-1, OxLDL, and atherosclerosis. Mediators Inflamm..

[34] Incalza M.A., D’Oria R., Natalicchio A., Perrini S., Laviola L., Giorgino F. (2018). Oxidative stress and reactive oxygen species in endothelial dysfunction associated with cardiovascular and metabolic diseases. Vascul Pharmacol..

[35] Bulua A.C., Simon A., Maddipati R., Pelletier M., Park H., Kim K.Y., Sack M.N., Kastner D.L., Siegel R.M. (2011). Mitochondrial reactive oxygen species promote production of proinflammatory cytokines and are elevated in TNFR1-associated periodic syndrome (TRAPS). J. Exp. Med..

[36] Mathews M.T., Berk B.C. (2008). PARP-1 inhibition prevents oxidative and nitrosative stress-induced endothelial cell death via transactivation of the VEGF receptor 2. Arterioscler. Thromb. Vasc. Biol..

[37] Radi R. (2004). Nitric oxide, oxidants, and protein tyrosine nitration. Proc. Natl. Acad. Sci. USA.

[38] Nolfi-Donegan D., Braganza A., Shiva S. (2020). Mitochondrial electron transport chain: Oxidative phosphorylation, oxidant production, and methods of measurement. Redox Biol..

[39] Zhao R.Z., Jiang S., Zhang L., Yu Z.B. (2019). Mitochondrial electron transport chain, ROS generation and uncoupling (Review). Int. J. Mol. Med..

[40] Houten S.M., Violante S., Ventura F.V., Wanders R.J. (2016). The Biochemistry and Physiology of Mitochondrial Fatty Acid beta-Oxidation and Its Genetic Disorders. Annu. Rev. Physiol..

[41] Adeva-Andany M.M., Carneiro-Freire N., Seco-Filgueira M., Fernandez-Fernandez C., Mourino-Bayolo D. (2019). Mitochondrial beta-oxidation of saturated fatty acids in humans. Mitochondrion.

[42] Schrauwen P., Schrauwen-Hinderling V., Hoeks J., Hesselink M.K. (2010). Mitochondrial dysfunction and lipotoxicity. Biochim. Biophys. Acta.

[43] Han H.J., Lee Y.J., Park S.H., Lee J.H., Taub M. (2005). High glucose-induced oxidative stress inhibits Na+/glucose cotransporter activity in renal proximal tubule cells. Am. J. Physiol. Renal Physiol..

[44] Li Y.X., Han T.T., Liu Y., Zheng S., Zhang Y., Liu W., Hu Y.M. (2015). Insulin resistance caused by lipotoxicity is related to oxidative stress and endoplasmic reticulum stress in LPL gene knockout heterozygous mice. Atherosclerosis.

[45] Tripathy D., Mohanty P., Dhindsa S., Syed T., Ghanim H., Aljada A., Dandona P. (2003). Elevation of Free Fatty Acids Induces Inflammation and Impairs Vascular Reactivity in Healthy Subjects. Diabetes.

[46] Kobayasi R., Akamine E.H., Davel A.P., Rodrigues M.A., Carvalho C.R., Rossoni L.V. (2010). Oxidative stress and inflammatory mediators contribute to endothelial dysfunction in high-fat diet-induced obesity in mice. J. Hypertens..

[47] Sun Y., Ge X., Li X., He J., Wei X., Du J., Sun J., Li X., Xun Z., Liu W. (2020). High-fat diet promotes renal injury by inducing oxidative stress and mitochondrial dysfunction. Cell Death Dis..

[48] Tangvarasittichai S. (2015). Oxidative stress, insulin resistance, dyslipidemia and type 2 diabetes mellitus. World J. Diabetes.

[49] Fouad Y., Waked I., Bollipo S., Gomaa A., Ajlouni Y., Attia D. (2020). What’s in a name? Renaming ‘NAFLD’ to ‘MAFLD’. Liver Int..

[50] Riazi K., Azhari H., Charette J.H., Underwood F.E., King J.A., Afshar E.E., Swain M.G., Congly S.E., Kaplan G.G., Shaheen A.A. (2022). The prevalence and incidence of NAFLD worldwide: A systematic review and meta-analysis. Lancet Gastroenterol. Hepatol..

[51] Teng M.L., Ng C.H., Huang D.Q., Chan K.E., Tan D.J., Lim W.H., Yang J.D., Tan E., Muthiah M.D. (2023). Global incidence and prevalence of nonalcoholic fatty liver disease. Clin. Mol. Hepatol..

[52] Eslam M., Sanyal A.J., George J., International Consensus P. (2020). MAFLD: A Consensus-Driven Proposed Nomenclature for Metabolic Associated Fatty Liver Disease. Gastroenterology.

[53] Chalasani N., Younossi Z., Lavine J.E., Charlton M., Cusi K., Rinella M., Harrison S.A., Brunt E.M., Sanyal A.J. (2018). The diagnosis and management of nonalcoholic fatty liver disease: Practice guidance from the American Association for the Study of Liver Diseases. Hepatology.

[54] Romero-Gomez M., Zelber-Sagi S., Trenell M. (2017). Treatment of NAFLD with diet, physical activity and exercise. J. Hepatol..

[55] Ryan M.C., Itsiopoulos C., Thodis T., Ward G., Trost N., Hofferberth S., O’Dea K., Desmond P.V., Johnson N.A., Wilson A.M. (2013). The Mediterranean diet improves hepatic steatosis and insulin sensitivity in individuals with non-alcoholic fatty liver disease. J. Hepatol..

[56] Hashida R., Kawaguchi T., Bekki M., Omoto M., Matsuse H., Nago T., Takano Y., Ueno T., Koga H., George J. (2017). Aerobic vs. resistance exercise in non-alcoholic fatty liver disease: A systematic review. J. Hepatol..

[57] Newsome P.N., Buchholtz K., Cusi K., Linder M., Okanoue T., Ratziu V., Sanyal A.J., Sejling A.S., Harrison S.A., Investigators N.N. (2021). A Placebo-Controlled Trial of Subcutaneous Semaglutide in Nonalcoholic Steatohepatitis. N. Engl. J. Med..

[58] Loomba R., Abdelmalek M.F., Armstrong M.J., Jara M., Kjær M.S., Krarup N., Lawitz E., Ratziu V., Sanyal A.J., Schattenberg J.M. (2023). Semaglutide 2·4 mg once weekly in patients with non-alcoholic steatohepatitis-related cirrhosis: A randomised, placebo-controlled phase 2 trial. Lancet.

[59] Baker N., Chaikoff I.L., Schusdek A. (1952). Effect of Fructose on Lipogenesis from Lactate and Acetate in Diabetic Liver. J. Biol. Chem..

[60] Li M.V., Chen W., Harmancey R.N., Nuotio-Antar A.M., Imamura M., Saha P., Taegtmeyer H., Chan L. (2010). Glucose-6-phosphate mediates activation of the carbohydrate responsive binding protein (ChREBP). Biochem. Biophys. Res. Commun..

[61] Softic S., Gupta M.K., Wang G.X., Fujisaka S., O’Neill B.T., Rao T.N., Willoughby J., Harbison C., Fitzgerald K., Ilkayeva O. (2017). Divergent effects of glucose and fructose on hepatic lipogenesis and insulin signaling. J. Clin. Investig..

[62] Zhao S., Jang C., Liu J., Uehara K., Gilbert M., Izzo L., Zeng X., Trefely S., Fernandez S., Carrer A. (2020). Dietary fructose feeds hepatic lipogenesis via microbiota-derived acetate. Nature.

[63] Morrow M.R., Batchuluun B., Wu J., Ahmadi E., Leroux J.M., Mohammadi-Shemirani P., Desjardins E.M., Wang Z., Tsakiridis E.E., Lavoie D.C.T. (2022). Inhibition of ATP-citrate lyase improves NASH, liver fibrosis, and dyslipidemia. Cell Metab..

[64] Cox C.L., Stanhope K.L., Schwarz J.M., Graham J.L., Hatcher B., Griffen S.C., Bremer A.A., Berglund L., McGahan J.P., Havel P.J. (2012). Consumption of fructose-sweetened beverages for 10 weeks reduces net fat oxidation and energy expenditure in overweight/obese men and women. Eur. J. Clin. Nutr..

[65] Softic S., Meyer J.G., Wang G.X., Gupta M.K., Batista T.M., Lauritzen H., Fujisaka S., Serra D., Herrero L., Willoughby J. (2019). Dietary Sugars Alter Hepatic Fatty Acid Oxidation via Transcriptional and Post-translational Modifications of Mitochondrial Proteins. Cell Metab..

[66] Cho Y.E., Kim D.K., Seo W., Gao B., Yoo S.H., Song B.J. (2021). Fructose Promotes Leaky Gut, Endotoxemia, and Liver Fibrosis Through Ethanol-Inducible Cytochrome P450-2E1-Mediated Oxidative and Nitrative Stress. Hepatology.

[67] Abdelmalek M.F., Suzuki A., Guy C., Unalp-Arida A., Colvin R., Johnson R.J., Diehl A.M., Nonalcoholic Steatohepatitis Clinical Research N. (2010). Increased fructose consumption is associated with fibrosis severity in patients with nonalcoholic fatty liver disease. Hepatology.

[68] Pérez-Carreras M., Del Hoyo P., Martín M.A., Rubio J.C., Martín A., Castellano G., Colina F., Arenas J., Solis-Herruzo J.A. (2003). Defective hepatic mitochondrial respiratory chain in patients with nonalcoholic steatohepatitis. Hepatology.

[69] Koek G.H., Liedorp P.R., Bast A. (2011). The role of oxidative stress in non-alcoholic steatohepatitis. Clin. Chim. Acta.

[70] Haufe S., Engeli S., Kast P., Bohnke J., Utz W., Haas V., Hermsdorf M., Mahler A., Wiesner S., Birkenfeld A.L. (2011). Randomized comparison of reduced fat and reduced carbohydrate hypocaloric diets on intrahepatic fat in overweight and obese human subjects. Hepatology.

[71] Westerbacka J., Lammi K., Hakkinen A.M., Rissanen A., Salminen I., Aro A., Yki-Jarvinen H. (2005). Dietary fat content modifies liver fat in overweight nondiabetic subjects. J. Clin. Endocrinol. Metab..

[72] Bozzetto L., Prinster A., Annuzzi G., Costagliola L., Mangione A., Vitelli A., Mazzarella R., Longobardo M., Mancini M., Vigorito C. (2012). Liver fat is reduced by an isoenergetic MUFA diet in a controlled randomized study in type 2 diabetic patients. Diabetes Care.

[73] Rezaei S., Akhlaghi M., Sasani M.R., Barati Boldaji R. (2019). Olive oil lessened fatty liver severity independent of cardiometabolic correction in patients with non-alcoholic fatty liver disease: A randomized clinical trial. Nutrition.

[74] Yang J., Fernandez-Galilea M., Martinez-Fernandez L., Gonzalez-Muniesa P., Perez-Chavez A., Martinez J.A., Moreno-Aliaga M.J. (2019). Oxidative Stress and Non-Alcoholic Fatty Liver Disease: Effects of Omega-3 Fatty Acid Supplementation. Nutrients.

[75] Oh D.Y., Walenta E., Akiyama T.E., Lagakos W.S., Lackey D., Pessentheiner A.R., Sasik R., Hah N., Chi T.J., Cox J.M. (2014). A Gpr120-selective agonist improves insulin resistance and chronic inflammation in obese mice. Nat. Med..

[76] Oh D.Y., Talukdar S., Bae E.J., Imamura T., Morinaga H., Fan W., Li P., Lu W.J., Watkins S.M., Olefsky J.M. (2010). GPR120 is an omega-3 fatty acid receptor mediating potent anti-inflammatory and insulin-sensitizing effects. Cell.

[77] Im D.S. (2012). Omega-3 fatty acids in anti-inflammation (pro-resolution) and GPCRs. Prog. Lipid Res..

[78] Serhan C.N. (2014). Pro-resolving lipid mediators are leads for resolution physiology. Nature.

[79] Casas R., Castro-Barquero S., Estruch R., Sacanella E. (2018). Nutrition and Cardiovascular Health. Int. J. Mol. Sci..

[80] Sanyal A.J., Abdelmalek M.F., Suzuki A., Cummings O.W., Chojkier M., Group E.-A.S. (2014). No significant effects of ethyl-eicosapentanoic acid on histologic features of nonalcoholic steatohepatitis in a phase 2 trial. Gastroenterology.

[81] Vega G.L., Chandalia M., Szczepaniak L.S., Grundy S.M. (2008). Effects of N-3 fatty acids on hepatic triglyceride content in humans. J. Investig. Med..

[82] Innes J.K., Calder P.C. (2018). Omega-6 fatty acids and inflammation. Prostaglandins Leukot. Essent. Fatty Acids.

[83] Sztolsztener K., Chabowski A., Harasim-Symbor E., Bielawiec P., Konstantynowicz-Nowicka K. (2020). Arachidonic Acid as an Early Indicator of Inflammation during Non-Alcoholic Fatty Liver Disease Development. Biomolecules.

[84] Bjermo H., Iggman D., Kullberg J., Dahlman I., Johansson L., Persson L., Berglund J., Pulkki K., Basu S., Uusitupa M. (2012). Effects of n-6 PUFAs compared with SFAs on liver fat, lipoproteins, and inflammation in abdominal obesity: A randomized controlled trial. Am. J. Clin. Nutr..

[85] Ferolla S.M., Ferrari T.C., Lima M.L., Reis T.O., Tavares W.C., Couto O.F., Vidigal P.V., Fausto M.A., Couto C.A. (2013). Dietary patterns in Brazilian patients with nonalcoholic fatty liver disease: A cross-sectional study. Clinics.

[86] Musso G., Gambino R., De Michieli F., Cassader M., Rizzetto M., Durazzo M., Faga E., Silli B., Pagano G. (2003). Dietary habits and their relations to insulin resistance and postprandial lipemia in nonalcoholic steatohepatitis. Hepatology.

[87] Utzschneider K.M., Bayer-Carter J.L., Arbuckle M.D., Tidwell J.M., Richards T.L., Craft S. (2013). Beneficial effect of a weight-stable, low-fat/low-saturated fat/low-glycaemic index diet to reduce liver fat in older subjects. Br. J. Nutr..

[88] Marina A., von Frankenberg A.D., Suvag S., Callahan H.S., Kratz M., Richards T.L., Utzschneider K.M. (2014). Effects of dietary fat and saturated fat content on liver fat and markers of oxidative stress in overweight/obese men and women under weight-stable conditions. Nutrients.

[89] Bortolotti M., Kreis R., Debard C., Cariou B., Faeh D., Chetiveaux M., Ith M., Vermathen P., Stefanoni N., Le K.A. (2009). High protein intake reduces intrahepatocellular lipid deposition in humans. Am. J. Clin. Nutr..

[90] Luukkonen P.K., Sadevirta S., Zhou Y., Kayser B., Ali A., Ahonen L., Lallukka S., Pelloux V., Gaggini M., Jian C. (2018). Saturated Fat Is More Metabolically Harmful for the Human Liver Than Unsaturated Fat or Simple Sugars. Diabetes Care.

[91] Jensen V.S., Hvid H., Damgaard J., Nygaard H., Ingvorsen C., Wulff E.M., Lykkesfeldt J., Fledelius C. (2018). Dietary fat stimulates development of NAFLD more potently than dietary fructose in Sprague-Dawley rats. Diabetol. Metab. Syndr..

[92] Romeo S., Kozlitina J., Xing C., Pertsemlidis A., Cox D., Pennacchio L.A., Boerwinkle E., Cohen J.C., Hobbs H.H. (2008). Genetic variation in PNPLA3 confers susceptibility to nonalcoholic fatty liver disease. Nat. Genet..

[93] He S., McPhaul C., Li J.Z., Garuti R., Kinch L., Grishin N.V., Cohen J.C., Hobbs H.H. (2010). A sequence variation (I148M) in PNPLA3 associated with nonalcoholic fatty liver disease disrupts triglyceride hydrolysis. J. Biol. Chem..

[94] Anstee Q.M., Day C.P. (2015). The Genetics of Nonalcoholic Fatty Liver Disease: Spotlight on PNPLA3 and TM6SF2. Semin. Liver Dis..

[95] Rotman Y., Koh C., Zmuda J.M., Kleiner D.E., Liang T.J., Nash C.R.N. (2010). The association of genetic variability in patatin-like phospholipase domain-containing protein 3 (PNPLA3) with histological severity of nonalcoholic fatty liver disease. Hepatology.

[96] Romeo S., Sentinelli F., Cambuli V.M., Incani M., Congiu T., Matta V., Pilia S., Huang-Doran I., Cossu E., Loche S. (2010). The 148M allele of the PNPLA3 gene is associated with indices of liver damage early in life. J. Hepatol..

[97] Shen J.H., Li Y.L., Li D., Wang N.N., Jing L., Huang Y.H. (2015). The rs738409 (I148M) variant of the PNPLA3 gene and cirrhosis: A meta-analysis. J. Lipid Res..

[98] Valenti L., Al-Serri A., Daly A.K., Galmozzi E., Rametta R., Dongiovanni P., Nobili V., Mozzi E., Roviaro G., Vanni E. (2010). Homozygosity for the patatin-like phospholipase-3/adiponutrin I148M polymorphism influences liver fibrosis in patients with nonalcoholic fatty liver disease. Hepatology.

[99] Nischalke H.D., Berger C., Luda C., Berg T., Muller T., Grunhage F., Lammert F., Coenen M., Kramer B., Korner C. (2011). The PNPLA3 rs738409 148M/M genotype is a risk factor for liver cancer in alcoholic cirrhosis but shows no or weak association in hepatitis C cirrhosis. PLoS ONE.

[100] Huang Y., Cohen J.C., Hobbs H.H. (2011). Expression and characterization of a PNPLA3 protein isoform (I148M) associated with nonalcoholic fatty liver disease. J. Biol. Chem..

[101] Lake A.C., Sun Y., Li J.L., Kim J.E., Johnson J.W., Li D., Revett T., Shih H.H., Liu W., Paulsen J.E. (2005). Expression, regulation, and triglyceride hydrolase activity of Adiponutrin family members. J. Lipid Res..

[102] Jenkins C.M., Mancuso D.J., Yan W., Sims H.F., Gibson B., Gross R.W. (2004). Identification, cloning, expression, and purification of three novel human calcium-independent phospholipase A2 family members possessing triacylglycerol lipase and acylglycerol transacylase activities. J. Biol. Chem..

[103] Kumari M., Schoiswohl G., Chitraju C., Paar M., Cornaciu I., Rangrez A.Y., Wongsiriroj N., Nagy H.M., Ivanova P.T., Scott S.A. (2012). Adiponutrin functions as a nutritionally regulated lysophosphatidic acid acyltransferase. Cell Metab..

[104] Yang A., Mottillo E.P., Mladenovic-Lucas L., Zhou L., Granneman J.G. (2019). Dynamic interactions of ABHD5 with PNPLA3 regulate triacylglycerol metabolism in brown adipocytes. Nat. Metab..

[105] Chen W., Chang B., Li L., Chan L. (2010). Patatin-like phospholipase domain-containing 3/adiponutrin deficiency in mice is not associated with fatty liver disease. Hepatology.

[106] Basantani M.K., Sitnick M.T., Cai L., Brenner D.S., Gardner N.P., Li J.Z., Schoiswohl G., Yang K., Kumari M., Gross R.W. (2011). Pnpla3/Adiponutrin deficiency in mice does not contribute to fatty liver disease or metabolic syndrome. J. Lipid Res..

[107] Li J.Z., Huang Y., Karaman R., Ivanova P.T., Brown H.A., Roddy T., Castro-Perez J., Cohen J.C., Hobbs H.H. (2012). Chronic overexpression of PNPLA3I148M in mouse liver causes hepatic steatosis. J. Clin. Investig..

[108] Smagris E., BasuRay S., Li J., Huang Y., Lai K.M., Gromada J., Cohen J.C., Hobbs H.H. (2015). Pnpla3I148M knockin mice accumulate PNPLA3 on lipid droplets and develop hepatic steatosis. Hepatology.

[109] Mitsche M.A., Hobbs H.H., Cohen J.C. (2018). Patatin-like phospholipase domain-containing protein 3 promotes transfer of essential fatty acids from triglycerides to phospholipids in hepatic lipid droplets. J. Biol. Chem..

[110] Kumashiro N., Yoshimura T., Cantley J.L., Majumdar S.K., Guebre-Egziabher F., Kursawe R., Vatner D.F., Fat I., Kahn M., Erion D.M. (2013). Role of patatin-like phospholipase domain-containing 3 on lipid-induced hepatic steatosis and insulin resistance in rats. Hepatology.

[111] Luukkonen P.K., Nick A., Holtta-Vuori M., Thiele C., Isokuortti E., Lallukka-Bruck S., Zhou Y., Hakkarainen A., Lundbom N., Peltonen M. (2019). Human PNPLA3-I148M variant increases hepatic retention of polyunsaturated fatty acids. JCI Insight.

[112] Tilson S.G., Morell C.M., Lenaerts A.S., Park S.B., Hu Z., Jenkins B., Koulman A., Liang T.J., Vallier L. (2021). Modeling PNPLA3-Associated NAFLD Using Human-Induced Pluripotent Stem Cells. Hepatology.

[113] Kabbani M., Michailidis E., Steensels S., Fulmer C.G., Luna J.M., Le Pen J., Tardelli M., Razooky B., Ricardo-Lax I., Zou C. (2022). Human hepatocyte PNPLA3-148M exacerbates rapid non-alcoholic fatty liver disease development in chimeric mice. Cell Rep..

[114] Wang Y., Kory N., BasuRay S., Cohen J.C., Hobbs H.H. (2019). PNPLA3, CGI-58, and Inhibition of Hepatic Triglyceride Hydrolysis in Mice. Hepatology.

[115] Sookoian S., Castano G.O., Scian R., Mallardi P., Fernandez Gianotti T., Burgueno A.L., San Martino J., Pirola C.J. (2015). Genetic variation in transmembrane 6 superfamily member 2 and the risk of nonalcoholic fatty liver disease and histological disease severity. Hepatology.

[116] Kozlitina J., Smagris E., Stender S., Nordestgaard B.G., Zhou H.H., Tybjaerg-Hansen A., Vogt T.F., Hobbs H.H., Cohen J.C. (2014). Exome-wide association study identifies a TM6SF2 variant that confers susceptibility to nonalcoholic fatty liver disease. Nat. Genet..

[117] Mahdessian H., Taxiarchis A., Popov S., Silveira A., Franco-Cereceda A., Hamsten A., Eriksson P., van’t Hooft F. (2014). TM6SF2 is a regulator of liver fat metabolism influencing triglyceride secretion and hepatic lipid droplet content. Proc. Natl. Acad. Sci. USA.

[118] Smagris E., Gilyard S., BasuRay S., Cohen J.C., Hobbs H.H. (2016). Inactivation of Tm6sf2, a Gene Defective in Fatty Liver Disease, Impairs Lipidation but Not Secretion of Very Low Density Lipoproteins. J. Biol. Chem..

[119] Luo F., Oldoni F., Das A. (2022). TM6SF2: A Novel Genetic Player in Nonalcoholic Fatty Liver and Cardiovascular Disease. Hepatol. Commun..

[120] Shindou H., Hishikawa D., Harayama T., Yuki K., Shimizu T. (2009). Recent progress on acyl CoA: Lysophospholipid acyltransferase research. J. Lipid Res..

[121] Shindou H., Shimizu T. (2009). Acyl-CoA:lysophospholipid acyltransferases. J. Biol. Chem..

[122] Xia M., Chandrasekaran P., Rong S., Fu X., Mitsche M.A. (2021). Hepatic deletion of Mboat7 (LPIAT1) causes activation of SREBP-1c and fatty liver. J. Lipid Res..

[123] Tanaka Y., Shimanaka Y., Caddeo A., Kubo T., Mao Y., Kubota T., Kubota N., Yamauchi T., Mancina R.M., Baselli G. (2021). LPIAT1/MBOAT7 depletion increases triglyceride synthesis fueled by high phosphatidylinositol turnover. Gut.

[124] Helsley R.N., Varadharajan V., Brown A.L., Gromovsky A.D., Schugar R.C., Ramachandiran I., Fung K., Kabbany M.N., Banerjee R., Neumann C.K. (2019). Obesity-linked suppression of membrane-bound O-acyltransferase 7 (MBOAT7) drives non-alcoholic fatty liver disease. eLife.

[125] Thangapandi V.R., Knittelfelder O., Brosch M., Patsenker E., Vvedenskaya O., Buch S., Hinz S., Hendricks A., Nati M., Herrmann A. (2021). Loss of hepatic Mboat7 leads to liver fibrosis. Gut.

[126] Sharpe M.C., Pyles K.D., Hallcox T., Kamm D.R., Piechowski M., Fisk B., Albert C.J., Carpenter D.H., Ulmasov B., Ford D.A. (2023). Enhancing Hepatic MBOAT7 Expression in Mice With Nonalcoholic Steatohepatitis. Gastro Hep Adv..

[127] Meroni M., Dongiovanni P., Longo M., Carli F., Baselli G., Rametta R., Pelusi S., Badiali S., Maggioni M., Gaggini M. (2020). Mboat7 down-regulation by hyper-insulinemia induces fat accumulation in hepatocytes. EBioMedicine.

[128] Alharthi J., Bayoumi A., Thabet K., Pan Z., Gloss B.S., Latchoumanin O., Lundberg M., Twine N.A., McLeod D., Alenizi S. (2022). A metabolic associated fatty liver disease risk variant in MBOAT7 regulates toll like receptor induced outcomes. Nat. Commun..

[129] Zhou R., Yazdi A.S., Menu P., Tschopp J. (2011). A role for mitochondria in NLRP3 inflammasome activation. Nature.

[130] West A.P., Brodsky I.E., Rahner C., Woo D.K., Erdjument-Bromage H., Tempst P., Walsh M.C., Choi Y., Shadel G.S., Ghosh S. (2011). TLR signalling augments macrophage bactericidal activity through mitochondrial ROS. Nature.

[131] Chen Y., Du X., Kuppa A., Feitosa M.F., Bielak L.F., O’Connell J.R., Musani S.K., Guo X., Kahali B., Chen V.L. (2023). Genome-wide association meta-analysis identifies 17 loci associated with nonalcoholic fatty liver disease. Nat. Genet..

[132] Stender S., Kozlitina J., Nordestgaard B.G., Tybjaerg-Hansen A., Hobbs H.H., Cohen J.C. (2017). Adiposity amplifies the genetic risk of fatty liver disease conferred by multiple loci. Nat. Genet..

[133] Huang Y., He S., Li J.Z., Seo Y.K., Osborne T.F., Cohen J.C., Hobbs H.H. (2010). A feed-forward loop amplifies nutritional regulation of PNPLA3. Proc. Natl. Acad. Sci. USA.

[134] Jones R.B., Arenaza L., Rios C., Plows J.F., Berger P.K., Alderete T.L., Fogel J.L., Nayak K., Mohamed P., Hwang D. (2021). PNPLA3 Genotype, Arachidonic Acid Intake, and Unsaturated Fat Intake Influences Liver Fibrosis in Hispanic Youth with Obesity. Nutrients.

[135] Van Name M.A., Savoye M., Chick J.M., Galuppo B.T., Feldstein A.E., Pierpont B., Johnson C., Shabanova V., Ekong U., Valentino P.L. (2020). A Low omega-6 to omega-3 PUFA Ratio (n-6:n-3 PUFA) Diet to Treat Fatty Liver Disease in Obese Youth. J. Nutr..

[136] Pirazzi C., Adiels M., Burza M.A., Mancina R.M., Levin M., Stahlman M., Taskinen M.R., Orho-Melander M., Perman J., Pujia A. (2012). Patatin-like phospholipase domain-containing 3 (PNPLA3) I148M (rs738409) affects hepatic VLDL secretion in humans and in vitro. J. Hepatol..

[137] Stojkovic I.A., Ericson U., Rukh G., Riddestrale M., Romeo S., Orho-Melander M. (2014). The PNPLA3 Ile148Met interacts with overweight and dietary intakes on fasting triglyceride levels. Genes Nutr..

[138] Simons N., Isaacs A., Koek G.H., Kuc S., Schaper N.C., Brouwers M. (2017). PNPLA3, TM6SF2, and MBOAT7 Genotypes and Coronary Artery Disease. Gastroenterology.

[139] Mancina R.M., Dongiovanni P., Petta S., Pingitore P., Meroni M., Rametta R., Boren J., Montalcini T., Pujia A., Wiklund O. (2016). The MBOAT7-TMC4 Variant rs641738 Increases Risk of Nonalcoholic Fatty Liver Disease in Individuals of European Descent. Gastroenterology.

[140] Krawczyk M., Jimenez-Aguero R., Alustiza J.M., Emparanza J.I., Perugorria M.J., Bujanda L., Lammert F., Banales J.M. (2016). PNPLA3 p.I148M variant is associated with greater reduction of liver fat content after bariatric surgery. Surg. Obes. Relat. Dis..

[141] Tabassum R., Ramo J.T., Ripatti P., Koskela J.T., Kurki M., Karjalainen J., Palta P., Hassan S., Nunez-Fontarnau J., Kiiskinen T.T.J. (2019). Genetic architecture of human plasma lipidome and its link to cardiovascular disease. Nat. Commun..

[142] Linton M.F., Yancey P.G., Davies S.S., Jerome W.G., Linton E.F., Song W.L., Doran A.C., Vickers K.C., Feingold K.R., Anawalt B., Blackman M.R., Boyce A., Chrousos G., Corpas E.K.R., Anawalt B., Blackman M.R., Boyce A., Chrousos G. (2000). The Role of Lipids and Lipoproteins in Atherosclerosis. Endotext [Internet].

[143] Hu F., Colditz G., Rosner B., Hennekens C., Willett W. (1997). Dietary Fat Intake and the Risk of Coronary Heart Disease in Women. N. Engl. J. Med..

[144] Schwingshackl L., Hoffmann G. (2014). Dietary fatty acids in the secondary prevention of coronary heart disease: A systematic review, meta-analysis and meta-regression. BMJ Open.

[145] Siri-Tarino P.W., Sun Q., Hu F.B., Krauss R.M. (2010). Meta-analysis of prospective cohort studies evaluating the association of saturated fat with cardiovascular disease. Am. J. Clin. Nutr..

[146] Hooper L., Martin N., Jimoh O.F., Kirk C., Foster E., Abdelhamid A.S. (2020). Reduction in saturated fat intake for cardiovascular disease. Cochrane Database Syst. Rev..

[147] Maki K.C., Dicklin M.R., Kirkpatrick C.F. (2021). Saturated fats and cardiovascular health: Current evidence and controversies. J. Clin. Lipidol..

[148] Cani P.D., Amar J., Iglesias M.A., Poggi M., Knauf C., Bastelica D., Neyrinck A.M., Fava F., Tuohy K.M., Chabo C. (2007). Metabolic endotoxemia initiates obesity and insulin resistance. Diabetes.

[149] Lin J., Yang R., Tarr P.T., Wu P.H., Handschin C., Li S., Yang W., Pei L., Uldry M., Tontonoz P. (2005). Hyperlipidemic effects of dietary saturated fats mediated through PGC-1beta coactivation of SREBP. Cell.

[150] Rudel L.P.J., Sawyer J. (1995). Compared With Dietary Monounsaturated and Saturated Fat, Polyunsaturated Fat Protects African Green Monkeys From Coronary Artery Atherosclerosis. Arterioscler. Thromb. Vase Biol..

[151] Ramsden C.E., Zamora D., Majchrzak-Hong S., Faurot K.R., Broste S.K., Frantz R.P., Davis J.M., Ringel A., Suchindran C.M., Hibbeln J.R. (2016). Re-evaluation of the traditional diet-heart hypothesis: Analysis of recovered data from Minnesota Coronary Experiment (1968-73). BMJ Open.

[152] Ramsden C.E., Zamora D., Leelarthaepin B., Majchrzak-Hong S.F., Faurot K.R., Suchindran C.M., Ringel A., Hibbeln J.R. (2013). Use of dietary linoleic acid for secondary prevention of coronary heart disease and death: Evaluation of recovered data from the Sydney Diet Heart Study and updated meta-analysis. FASEB.

[153] Leren P. (1968). The effect of plasma-cholesterol-lowering diet in male survivors of myocardial infarction. A controlled clinical trial. Bull. N. Y. Acad. Med..

[154] Wang Y., Fang Y., Witting P.K., Charchar F.J., Sobey C.G., Drummond G.R., Golledge J. (2023). Dietary fatty acids and mortality risk from heart disease in US adults: An analysis based on NHANES. Sci. Rep..

[155] Dayton S., Pearce M.L., Goldman H., Harnish A., Plotkin D., Shickman M., Winfield M., Zager A., Dixon W. (1968). Controlled trial of a diet high in unsaturated fat for prevention of atherosclerotic complications. Lancet.

[156] Karvonen M.J., Pekkarinen M., Miettinen M., Elosuo R., Paavilainen E., Paavilainen E. (1979). Dietary prevention of coronary heart disease: The Finnish Mental Hospital Study. Int. J. Epidemiol..

[157] Sacks F.M., Lichtenstein A.H., Wu J.H.Y., Appel L.J., Creager M.A., Kris-Etherton P.M., Miller M., Rimm E.B., Rudel L.L., Robinson J.G. (2017). Dietary Fats and Cardiovascular Disease: A Presidential Advisory From the American Heart Association. Circulation.

[158] Jakobsen M.U., O’Reilly E.J., Heitmann B.L., Pereira M.A., Balter K., Fraser G.E., Goldbourt U., Hallmans G., Knekt P., Liu S. (2009). Major types of dietary fat and risk of coronary heart disease: A pooled analysis of 11 cohort studies. Am. J. Clin. Nutr..

[159] Wang Q., Liang X., Wang L., Lu X., Huang J., Cao J., Li H., Gu D. (2012). Effect of omega-3 fatty acids supplementation on endothelial function: A meta-analysis of randomized controlled trials. Atherosclerosis.

[160] Serhan C.N., Hong S., Gronert K., Colgan S.P., Devchand P.R., Mirick G., Moussignac R.L. (2002). Resolvins: A family of bioactive products of omega-3 fatty acid transformation circuits initiated by aspirin treatment that counter proinflammation signals. J. Exp. Med..

[161] Heydari B., Abdullah S., Pottala J.V., Shah R., Abbasi S., Mandry D., Francis S.A., Lumish H., Ghoshhajra B.B., Hoffmann U. (2016). Effect of Omega-3 Acid Ethyl Esters on Left Ventricular Remodeling After Acute Myocardial Infarction: The OMEGA-REMODEL Randomized Clinical Trial. Circulation.

[162] Simopoulos A.P. (2008). The importance of the omega-6/omega-3 fatty acid ratio in cardiovascular disease and other chronic diseases. Exp. Biol. Med..

[163] Parthasarathy S., Khoo J.C., Miller E., Barnett J., Witztum J.L., Steinberg D. (1990). Low density lipoprotein rich in oleic acid is protected against oxidative modification: Implications for dietary prevention of atherosclerosis. Proc. Natl. Acad. Sci. USA.

[164] Reaven P.D., Grasse B.J., Tribble D.L. (1994). Effects of linoleate-enriched and oleate-enriched diets in combination with alpha-tocopherol on the susceptibility of LDL and LDL subfractions to oxidative modification in humans. Arterioscler. Thromb..

[165] Louheranta A.M., Porkkala-Sarataho E.K., Nyyssonen M.K., Salonen R.M., Salonen J.T. (1996). Linoleic acid intake and susceptibility of very-low-density and low density lipoproteins to oxidation in men. Am. J. Clin. Nutr..

[166] Regnstrom J., Nilsson J., Tornvall P., Hamsten A., Landou C. (1992). Susceptibility to low-density lipoprotein oxidation and coronary atherosclerosis in man. Lancet.

[167] Mariamenatu A.H., Abdu E.M., Kostner G.M. (2021). Overconsumption of Omega-6 Polyunsaturated Fatty Acids (PUFAs) versus Deficiency of Omega-3 PUFAs in Modern-Day Diets: The Disturbing Factor for Their “Balanced Antagonistic Metabolic Functions” in the Human Body. J. Lipids.

[168] Fredman G., Hellmann J., Proto J.D., Kuriakose G., Colas R.A., Dorweiler B., Connolly E.S., Solomon R., Jones D.M., Heyer E.J. (2016). An imbalance between specialized pro-resolving lipid mediators and pro-inflammatory leukotrienes promotes instability of atherosclerotic plaques. Nat. Commun..

[169] Viola J.R., Lemnitzer P., Jansen Y., Csaba G., Winter C., Neideck C., Silvestre-Roig C., Dittmar G., Doring Y., Drechsler M. (2016). Resolving Lipid Mediators Maresin 1 and Resolvin D2 Prevent Atheroprogression in Mice. Circ. Res..

[170] Edwards-Glenn J.M., Fontes M.T., Waigi E.W., Costa T.J., Maiseyeu A., Webb R.C., McCarthy C.G., Wenceslau C.F. (2023). Specialized Pro-resolving Mediator Improves Vascular Relaxation via Formyl Peptide Receptor-2. Am. J. Hypertens..

[171] Massaro M., Habib A., Lubrano L., Del Turco S., Lazzerini G., Bourcier T., Weksler B.B., De Caterina R. (2006). The omega-3 fatty acid docosahexaenoate attenuates endothelial cyclooxygenase-2 induction through both NADP(H) oxidase and PKC epsilon inhibition. Proc. Natl. Acad. Sci. USA.

[172] Emken E.A., Rohwedder W.K., Adlof R.O., Rakoff H., Gulley R.M. (1987). Metabolism in humans ofcis-12,rans-15-octadecadienoic acid relative to palmitic, stearic, oleic and linoleic acids. Lipids.

[173] Indu M. (1992). Ghafoorunissa. n-3 fatty acids in Indian diets—Comparison of the effects of precursor (alpha-linolenic acid) Vs product (long chain n-3 poly unsaturated fatty acids). Nutr. Res..

[174] Yam D., Berry E.M., Berry E.M. (1996). Diet and disease--the Israeli paradox: Possible dangers of a high omega-6 polyunsaturated fatty acid diet. Isr. J. Med. Sci..

[175] Zhao Z.W., Zhang M., Zou J., Wan X.J., Zhou L., Wu Y., Liu S.M., Liao L.X., Li H., Qin Y.S. (2021). TIGAR mitigates atherosclerosis by promoting cholesterol efflux from macrophages. Atherosclerosis.

[176] Harris W.S., Mozaffarian D., Rimm E., Kris-Etherton P., Rudel L.L., Appel L.J., Engler M.M., Engler M.B., Sacks F. (2009). Omega-6 fatty acids and risk for cardiovascular disease: A science advisory from the American Heart Association Nutrition Subcommittee of the Council on Nutrition, Physical Activity, and Metabolism; Council on Cardiovascular Nursing; and Council on Epidemiology and Prevention. Circulation.

[177] Yoo S., Ahn H., Park Y.K. (2016). High Dietary Fructose Intake on Cardiovascular Disease Related Parameters in Growing Rats. Nutrients.

[178] Malik V.S., Popkin B.M., Bray G.A., Despres J.P., Willett W.C., Hu F.B. (2010). Sugar-sweetened beverages and risk of metabolic syndrome and type 2 diabetes: A meta-analysis. Diabetes Care.

[179] Kritchevsky D. (1975). The effects of feeding various carbohydrates on the development of hypercholesterolemia and atherosclerosis. Adv. Exp. Med. Biol..

[180] Gugliucci A., Lustig R.H., Caccavello R., Erkin-Cakmak A., Noworolski S.M., Tai V.W., Wen M.J., Mulligan K., Schwarz J.M. (2016). Short-term isocaloric fructose restriction lowers apoC-III levels and yields less atherogenic lipoprotein profiles in children with obesity and metabolic syndrome. Atherosclerosis.

[181] Tokita Y., Hirayama Y., Sekikawa A., Kotake H., Toyota T., Miyazawa T., Sawai T., Oikawa S. (2005). Fructose Ingestion Enhances Atherosclerosis and Deposition of Advanced Glycated End-products in Cholesterol-fed Rabbits. J. Atheroscler. Thromb..

[182] Chaurasia B., Tippetts T.S., Mayoral Monibas R., Liu J., Li Y., Wang L., Wilkerson J.L., Sweeney C.R., Pereira R.F., Sumida D.H. (2019). Targeting a ceramide double bond improves insulin resistance and hepatic steatosis. Science.

[183] Olson E., Suh J.H., Schwarz J.M., Noworolski S.M., Jones G.M., Barber J.R., Erkin-Cakmak A., Mulligan K., Lustig R.H., Mietus-Snyder M. (2022). Effects of Isocaloric Fructose Restriction on Ceramide Levels in Children with Obesity and Cardiometabolic Risk: Relation to Hepatic De Novo Lipogenesis and Insulin Sensitivity. Nutrients.

[184] Lian Y.G., Zhao H.Y., Wang S.J., Xu Q.L., Xia X.J. (2017). NLRP4 is an essential negative regulator of fructose-induced cardiac injury in vitro and in vivo. Biomed. Pharmacother..

[185] Wang X., Xu Z., Chang R., Zeng C., Zhao Y. (2023). High-Fructose Diet Induces Cardiac Dysfunction via Macrophage Recruitment in Adult Mice. J. Cardiovasc. Pharmacol. Ther..

[186] De Angelis K., Senador D.D., Mostarda C., Irigoyen M.C., Morris M. (2012). Sympathetic overactivity precedes metabolic dysfunction in a fructose model of glucose intolerance in mice. Am. J. Physiol. Regul. Integr. Comp. Physiol..

[187] Cannizzo B., Lujan A., Estrella N., Lembo C., Cruzado M., Castro C. (2012). Insulin resistance promotes early atherosclerosis via increased proinflammatory proteins and oxidative stress in fructose-fed ApoE-KO mice. Exp. Diabetes Res..

[188] Bensaad K., Tsuruta A., Selak M.A., Vidal M.N., Nakano K., Bartrons R., Gottlieb E., Vousden K.H. (2006). TIGAR, a p53-inducible regulator of glycolysis and apoptosis. Cell.

[189] Nikpay M., Goel A., Won H.H., Hall L.M., Willenborg C., Kanoni S., Saleheen D., Kyriakou T., Nelson C.P., Hopewell J.C. (2015). A comprehensive 1,000 Genomes-based genome-wide association meta-analysis of coronary artery disease. Nat. Genet..

[190] Utermann G., Hees M., Steinmetz A. (1977). Polymorphism of apolipoprotein E and occurrence of dysbetalipoproteinaemia in man. Nature.

[191] Carrasquilla G.D., Christiansen M.R., Kilpelainen T.O. (2021). The Genetic Basis of Hypertriglyceridemia. Curr. Atheroscler. Rep..

[192] Boren J., Chapman M.J., Krauss R.M., Packard C.J., Bentzon J.F., Binder C.J., Daemen M.J., Demer L.L., Hegele R.A., Nicholls S.J. (2020). Low-density lipoproteins cause atherosclerotic cardiovascular disease: Pathophysiological, genetic, and therapeutic insights: A consensus statement from the European Atherosclerosis Society Consensus Panel. Eur. Heart J..

[193] Zhang D.W., Lagace T.A., Garuti R., Zhao Z., McDonald M., Horton J.D., Cohen J.C., Hobbs H.H. (2007). Binding of proprotein convertase subtilisin/kexin type 9 to epidermal growth factor-like repeat A of low density lipoprotein receptor decreases receptor recycling and increases degradation. J. Biol. Chem..

[194] Meng F.H., Liu S., Xiao J., Zhou Y.X., Dong L.W., Li Y.F., Zhang Y.Q., Li W.H., Wang J.Q., Wang Y. (2023). New Loss-of-Function Mutations in PCSK9 Reduce Plasma LDL Cholesterol. Arterioscler. Thromb. Vasc. Biol..

[195] Orringer C.E., Jacobson T.A., Saseen J.J., Brown A.S., Gotto A.M., Ross J.L., Underberg J.A. (2017). Update on the use of PCSK9 inhibitors in adults: Recommendations from an Expert Panel of the National Lipid Association. J. Clin. Lipidol..

[196] Webster A.C., Nagler E.V., Morton R.L., Masson P. (2017). Chronic Kidney Disease. Lancet.

[197] Tonelli M., Wiebe N., Culleton B., House A., Rabbat C., Fok M., McAlister F., Garg A.X. (2006). Chronic kidney disease and mortality risk: A systematic review. J. Am. Soc. Nephrol..

[198] Romagnani P., Remuzzi G., Glassock R., Levin A., Jager K.J., Tonelli M., Massy Z., Wanner C., Anders H.J. (2017). Chronic kidney disease. Nat. Rev. Dis. Primers.

[199] Wuttke M., Li Y., Li M., Sieber K.B., Feitosa M.F., Gorski M., Tin A., Wang L., Chu A.Y., Hoppmann A. (2019). A catalog of genetic loci associated with kidney function from analyses of a million individuals. Nat. Genet..

[200] Rutledge J.C., Ng K.F., Aung H.H., Wilson D.W. (2010). Role of triglyceride-rich lipoproteins in diabetic nephropathy. Nat. Rev. Nephrol..

[201] van Herpen N.A., Schrauwen-Hinderling V.B. (2008). Lipid accumulation in non-adipose tissue and lipotoxicity. Physiol. Behav..

[202] Rada P., Gonzalez-Rodriguez A., Garcia-Monzon C., Valverde A.M. (2020). Understanding lipotoxicity in NAFLD pathogenesis: Is CD36 a key driver?. Cell Death Dis..

[203] Moorhead J.F., Chan M.K., El-Nahas M., Varghese Z. (1982). Lipid nephrotoxicity in chronic progressive glomerular and tubulo-interstitial disease. Lancet.

[204] Sharma M., Singh V., Sharma R., Koul A., McCarthy E.T., Savin V.J., Joshi T., Srivastava T. (2022). Glomerular Biomechanical Stress and Lipid Mediators during Cellular Changes Leading to Chronic Kidney Disease. Biomedicines.

[205] Badr K.F., Lakkis F.G. (1994). Lipoxygenase products in normal and diseased glomeruli. Ann. N. Y. Acad. Sci..

[206] McCarthy E.T., Sharma R., Sharma M. (2005). Protective effect of 20-hydroxyeicosatetraenoic acid (20-HETE) on glomerular protein permeability barrier. Kidney Int..

[207] Sharma M., McCarthy E.T., Reddy D.S., Patel P.K., Savin V.J., Medhora M., Falck J.R. (2009). 8,9-Epoxyeicosatrienoic acid protects the glomerular filtration barrier. Prostaglandins Other Lipid Mediat..

[208] Bobulescu I.A. (2010). Renal lipid metabolism and lipotoxicity. Curr. Opin. Nephrol. Hypertens..

[209] Xu Z.E., Chen Y., Huang A., Varghese Z., Moorhead J.F., Yan F., Powis S.H., Li Q., Ruan X.Z. (2011). Inflammatory stress exacerbates lipid-mediated renal injury in ApoE/CD36/SRA triple knockout mice. Am. J. Physiol. Renal Physiol..

[210] Rinaldi A., Lazareth H., Poindessous V., Nemazanyy I., Sampaio J.L., Malpetti D., Bignon Y., Naesens M., Rabant M., Anglicheau D. (2022). Impaired fatty acid metabolism perpetuates lipotoxicity along the transition to chronic kidney injury. JCI Insight.

[211] Hansell P., Welch W.J., Blantz R.C., Palm F. (2013). Determinants of kidney oxygen consumption and their relationship to tissue oxygen tension in diabetes and hypertension. Clin. Exp. Pharmacol. Physiol..

[212] Kang H.M., Ahn S.H., Choi P., Ko Y.A., Han S.H., Chinga F., Park A.S., Tao J., Sharma K., Pullman J. (2015). Defective fatty acid oxidation in renal tubular epithelial cells has a key role in kidney fibrosis development. Nat. Med..

[213] Hoeks J., Hesselink M.K., Russell A.P., Mensink M., Saris W.H., Mensink R.P., Schrauwen P. (2006). Peroxisome proliferator-activated receptor-gamma coactivator-1 and insulin resistance: Acute effect of fatty acids. Diabetologia.

[214] Koyama T., Kume S., Koya D., Araki S., Isshiki K., Chin-Kanasaki M., Sugimoto T., Haneda M., Sugaya T., Kashiwagi A. (2011). SIRT3 attenuates palmitate-induced ROS production and inflammation in proximal tubular cells. Free Radic. Biol. Med..

[215] Onodera T., Wang M.Y., Rutkowski J.M., Deja S., Chen S., Balzer M.S., Kim D.S., Sun X., An Y.A., Field B.C. (2023). Endogenous renal adiponectin drives gluconeogenesis through enhancing pyruvate and fatty acid utilization. Nat. Commun..

[216] Brinkkoetter P.T., Bork T., Salou S., Liang W., Mizi A., Ozel C., Koehler S., Hagmann H.H., Ising C., Kuczkowski A. (2019). Anaerobic Glycolysis Maintains the Glomerular Filtration Barrier Independent of Mitochondrial Metabolism and Dynamics. Cell Rep..

[217] Lee E., Choi J., Lee H.S. (2017). Palmitate induces mitochondrial superoxide generation and activates AMPK in podocytes. J. Cell Physiol..

[218] Xu S., Nam S.M., Kim J.H., Das R., Choi S.K., Nguyen T.T., Quan X., Choi S.J., Chung C.H., Lee E.Y. (2015). Palmitate induces ER calcium depletion and apoptosis in mouse podocytes subsequent to mitochondrial oxidative stress. Cell Death Dis..

[219] Lennon R., Pons D., Sabin M.A., Wei C., Shield J.P., Coward R.J., Tavare J.M., Mathieson P.W., Saleem M.A., Welsh G.I. (2009). Saturated fatty acids induce insulin resistance in human podocytes: Implications for diabetic nephropathy. Nephrol. Dial. Transplant..

[220] Tan B.L., Norhaizan M.E., Liew W.P. (2018). Nutrients and Oxidative Stress: Friend or Foe?. Oxid. Med. Cell Longev..

[221] Neurohr J.M., Paulson E.T., Kinsey S.T. (2021). A higher mitochondrial content is associated with greater oxidative damage, oxidative defenses, protein synthesis and ATP turnover in resting skeletal muscle. J. Exp. Biol..

[222] Opazo-Rios L., Mas S., Marin-Royo G., Mezzano S., Gomez-Guerrero C., Moreno J.A., Egido J. (2020). Lipotoxicity and Diabetic Nephropathy: Novel Mechanistic Insights and Therapeutic Opportunities. Int. J. Mol. Sci..

[223] Lanaspa M.A., Ishimoto T., Cicerchi C., Tamura Y., Roncal-Jimenez C.A., Chen W., Tanabe K., Andres-Hernando A., Orlicky D.J., Finol E. (2014). Endogenous fructose production and fructokinase activation mediate renal injury in diabetic nephropathy. J. Am. Soc. Nephrol..

[224] Hu Z., Ren L., Wang C., Liu B., Song G. (2012). Effect of chenodeoxycholic acid on fibrosis, inflammation and oxidative stress in kidney in high-fructose-fed Wistar rats. Kidney Blood Press. Res..

[225] Li Q., Xu Q., Tan J., Hu L., Ge C., Xu M. (2021). Carminic acid supplementation protects against fructose-induced kidney injury mainly through suppressing inflammation and oxidative stress via improving Nrf-2 signaling. Aging.

[226] Gherghina M.E., Peride I., Tiglis M., Neagu T.P., Niculae A., Checherita I.A. (2022). Uric Acid and Oxidative Stress-Relationship with Cardiovascular, Metabolic, and Renal Impairment. Int. J. Mol. Sci..

[227] Roncal C.A., Mu W., Croker B., Reungjui S., Ouyang X., Tabah-Fisch I., Johnson R.J., Ejaz A.A. (2007). Effect of elevated serum uric acid on cisplatin-induced acute renal failure. Am. J. Physiol. Renal Physiol..

[228] Lytvyn Y., Perkins B.A., Cherney D.Z. (2015). Uric acid as a biomarker and a therapeutic target in diabetes. Can. J. Diabetes.

[229] Abuja P.M. (1999). Ascorbate prevents prooxidant effects of urate in oxidation of human low density lipoprotein. FEBS Lett..

[230] Zharikov S., Krotova K., Hu H., Baylis C., Johnson R.J., Block E.R., Patel J. (2008). Uric acid decreases NO production and increases arginase activity in cultured pulmonary artery endothelial cells. Am. J. Physiol. Cell Physiol..

[231] Yu T., Robotham J.L., Yoon Y. (2006). Increased production of reactive oxygen species in hyperglycemic conditions requires dynamic change of mitochondrial morphology. Proc. Natl. Acad. Sci. USA.

[232] Nakagawa T., Johnson R.J., Andres-Hernando A., Roncal-Jimenez C., Sanchez-Lozada L.G., Tolan D.R., Lanaspa M.A. (2020). Fructose Production and Metabolism in the Kidney. J. Am. Soc. Nephrol..

[233] Lanaspa M.A., Sanchez-Lozada L.G., Cicerchi C., Li N., Roncal-Jimenez C.A., Ishimoto T., Le M., Garcia G.E., Thomas J.B., Rivard C.J. (2012). Uric acid stimulates fructokinase and accelerates fructose metabolism in the development of fatty liver. PLoS ONE.

[234] Lanaspa M.A., Sanchez-Lozada L.G., Choi Y.J., Cicerchi C., Kanbay M., Roncal-Jimenez C.A., Ishimoto T., Li N., Marek G., Duranay M. (2012). Uric acid induces hepatic steatosis by generation of mitochondrial oxidative stress: Potential role in fructose-dependent and -independent fatty liver. J. Biol. Chem..

[235] Gansevoort R.T., Matsushita K., van der Velde M., Astor B.C., Woodward M., Levey A.S., de Jong P.E., Coresh J., Chronic Kidney Disease Prognosis C. (2011). Lower estimated GFR and higher albuminuria are associated with adverse kidney outcomes. A collaborative meta-analysis of general and high-risk population cohorts. Kidney Int..

[236] Astor B.C., Matsushita K., Gansevoort R.T., van der Velde M., Woodward M., Levey A.S., Jong P.E., Coresh J., Chronic Kidney Disease Prognosis C., Astor B.C. (2011). Lower estimated glomerular filtration rate and higher albuminuria are associated with mortality and end-stage renal disease. A collaborative meta-analysis of kidney disease population cohorts. Kidney Int..

[237] Liu W.J., Xu B.H., Ye L., Liang D., Wu H.L., Zheng Y.Y., Deng J.K., Li B., Liu H.F. (2015). Urinary proteins induce lysosomal membrane permeabilization and lysosomal dysfunction in renal tubular epithelial cells. Am. J. Physiol. Renal Physiol..

[238] Nolin A.C., Mulhern R.M., Panchenko M.V., Pisarek-Horowitz A., Wang Z., Shirihai O., Borkan S.C., Havasi A. (2016). Proteinuria causes dysfunctional autophagy in the proximal tubule. Am. J. Physiol. Renal Physiol..

[239] Cybulsky A.V. (2017). Endoplasmic reticulum stress, the unfolded protein response and autophagy in kidney diseases. Nat. Rev. Nephrol..

[240] Abbate M., Zoja C., Remuzzi G. (2006). How does proteinuria cause progressive renal damage?. J. Am. Soc. Nephrol..

[241] Westenfelder C., Gooch A. (2022). Heme Protein-Induced Acute Kidney Injury Is Caused by Disruption of Mitochondrial Homeostasis in Proximal Tubular Cells. Kidney360.

[242] Zager R.A. (1996). Rhabdomyolysis and myohemoglobinuric acute renal failure. Kidney Int..

[243] Nath K.A., Singh R.D., Croatt A.J., Adams C.M. (2022). Heme Proteins and Kidney Injury: Beyond Rhabdomyolysis. Kidney360.

[244] Deng J.K., Zhang X., Wu H.L., Gan Y., Ye L., Zheng H., Zhu Z., Liu W.J., Liu H.F. (2021). ROS-ERK Pathway as Dual Mediators of Cellular Injury and Autophagy-Associated Adaptive Response in Urinary Protein-Irritated Renal Tubular Epithelial Cells. J. Diabetes Res..

[245] Kao W.H., Klag M.J., Meoni L.A., Reich D., Berthier-Schaad Y., Li M., Coresh J., Patterson N., Tandon A., Powe N.R. (2008). MYH9 is associated with nondiabetic end-stage renal disease in African Americans. Nat. Genet..

[246] Kottgen A., Glazer N.L., Dehghan A., Hwang S.J., Katz R., Li M., Yang Q., Gudnason V., Launer L.J., Harris T.B. (2009). Multiple loci associated with indices of renal function and chronic kidney disease. Nat. Genet..

[247] McKnight A.J., Currie D., Maxwell A.P. (2010). Unravelling the genetic basis of renal diseases; from single gene to multifactorial disorders. J. Pathol..

[248] Marzuillo P., Di Sessa A., Guarino S., Capalbo D., Umano G.R., Pedulla M., La Manna A., Cirillo G., Miraglia Del Giudice E. (2019). Nonalcoholic fatty liver disease and eGFR levels could be linked by the PNPLA3 I148M polymorphism in children with obesity. Pediatr. Obes..

[249] Di Sessa A., Russo M.C., Arienzo M.R., Umano G.R., Cozzolino D., Cirillo G., Guarino S., Miraglia Del Giudice E., Marzuillo P. (2022). PNPLA3 I148M Polymorphism Influences Renal Function in Children With Obesity and Prediabetes. J. Ren. Nutr..

[250] Verma S.B., Mittal A., Wollina U., Eckstein G.H., Gohel K., Giehl K. (2017). Chanarin-Dorfman syndrome with rare renal involvement. Br. J. Dermatol..

[251] Mantovani A., Zusi C., Sani E., Colecchia A., Lippi G., Zaza G.L., Valenti L., Byrne C.D., Maffeis C., Bonora E. (2019). Association between PNPLA3rs738409 polymorphism decreased kidney function in postmenopausal type 2 diabetic women with or without non-alcoholic fatty liver disease. Diabetes Metab..

[252] Zhao J., Rui H.L., Yang M., Sun L.J., Dong H.R., Cheng H. (2019). CD36-Mediated Lipid Accumulation and Activation of NLRP3 Inflammasome Lead to Podocyte Injury in Obesity-Related Glomerulopathy. Mediators Inflamm..

[253] Koo B.K., An J.N., Joo S.K., Kim D., Lee S., Bae J.M., Park J.H., Kim J.H., Chang M.S., Kim W. (2020). Association Between a Polymorphism in MBOAT7 and Chronic Kidney Disease in Patients With Biopsy-Confirmed Nonalcoholic Fatty Liver Disease. Clin. Gastroenterol. Hepatol..

[254] Varadharajan V., Massey W.J., Brown J.M. (2022). Membrane-bound O-acyltransferase 7 (MBOAT7)-driven phosphatidylinositol remodeling in advanced liver disease. J. Lipid Res..

[255] Musso G., Cassader M., Gambino R. (2015). PNPLA3 rs738409 and TM6SF2 rs58542926 gene variants affect renal disease and function in nonalcoholic fatty liver disease. Hepatology.

[256] Di Sessa A., Guarino S., Umano G.R., Arenella M., Alfiero S., Quaranta G., Miraglia Del Giudice E., Marzuillo P. (2021). MAFLD in Obese Children: A Challenging Definition. Children.

[257] Ma L., Wang S., Zhao H., Yu M., Deng X., Jiang Y., Cao Y., Li P., Niu W. (2021). Susceptibility of ApoB and PCSK9 Genetic Polymorphisms to Diabetic Kidney Disease Among Chinese Diabetic Patients. Front. Med..

[258] Xu Y., Liu B., Lin L., Lei F., Sun T., Zhang X., Song X., Huang X., Zeng Q., Cai J. (2023). The association of apolipoprotein B with chronic kidney disease in the Chinese population. Front. Endocrinol..

[259] Emanuelsson F., Nordestgaard B.G., Benn M. (2018). Familial Hypercholesterolemia and Risk of Peripheral Arterial Disease and Chronic Kidney Disease. J. Clin. Endocrinol. Metab..

[260] Vaseghi G., Javanmard S.H., Heshmat-Ghahdarijani K., Sarrafzadegan N., Amerizadeh A. (2023). Comorbidities with Familial Hypercholesterolemia (FH): A Systematic Review. Curr. Probl. Cardiol..

[261] Barbagallo C.M., Cefalu A.B., Giammanco A., Noto D., Caldarella R., Ciaccio M., Averna M.R., Nardi E. (2021). Lipoprotein Abnormalities in Chronic Kidney Disease and Renal Transplantation. Life.

[262] Ge S., Hertel B., Koltsova E.K., Sorensen-Zender I., Kielstein J.T., Ley K., Haller H., von Vietinghoff S. (2013). Increased atherosclerotic lesion formation and vascular leukocyte accumulation in renal impairment are mediated by interleukin-17A. Circ. Res..

[263] Targher G., Chonchol M.B., Byrne C.D. (2014). CKD and nonalcoholic fatty liver disease. Am. J. Kidney Dis..

[264] Musso G., Gambino R., Tabibian J.H., Ekstedt M., Kechagias S., Hamaguchi M., Hultcrantz R., Hagström H., Yoon S.K., Charatcharoenwitthaya P. (2014). Association of Non-alcoholic Fatty Liver Disease with Chronic Kidney Disease: A Systematic Review and Meta-analysis. PLoS Med..

[265] Targher G., Mantovani A., Alisi A., Mosca A., Panera N., Byrne C.D., Nobili V. (2019). Relationship Between PNPLA3 rs738409 Polymorphism and Decreased Kidney Function in Children with NAFLD. Hepatology.

[266] Huh J.H., Kim J.Y., Choi E., Kim J.S., Chang Y., Sung K.C. (2017). The fatty liver index as a predictor of incident chronic kidney disease in a 10-year prospective cohort study. PLoS ONE.

[267] Jang H.R., Kang D., Sinn D.H., Gu S., Cho S.J., Lee J.E., Huh W., Paik S.W., Ryu S., Chang Y. (2018). Nonalcoholic fatty liver disease accelerates kidney function decline in patients with chronic kidney disease: A cohort study. Sci. Rep..

[268] Onnerhag K., Dreja K., Nilsson P.M., Lindgren S. (2019). Increased mortality in non-alcoholic fatty liver disease with chronic kidney disease is explained by metabolic comorbidities. Clin. Res. Hepatol. Gastroenterol..

[269] Park H., Dawwas G.K., Liu X., Nguyen M.H. (2019). Nonalcoholic fatty liver disease increases risk of incident advanced chronic kidney disease: A propensity-matched cohort study. J. Intern. Med..

[270] Mantovani A., Zaza G., Byrne C.D., Lonardo A., Zoppini G., Bonora E., Targher G. (2018). Nonalcoholic fatty liver disease increases risk of incident chronic kidney disease: A systematic review and meta-analysis. Metabolism.

[271] Marcuccilli M., Chonchol M. (2016). NAFLD and Chronic Kidney Disease. Int. J. Mol. Sci..

[272] Massiera F., Bloch-Faure M., Ceiler D., Murakami K., Fukamizu A., Gasc J.M., Quignard-Boulange A., Negrel R., Ailhaud G., Seydoux J. (2001). Adipose angiotensinogen is involved in adipose tissue growth and blood pressure regulation. FASEB J..

[273] Frederich R., Kahn B., Peach M., Flier J. (1992). Tissue-specific nutritional regulation of angiotensinogen in adipose tissue. Hypertension.

[274] Pahlavani M., Kalupahana N.S., Ramalingam L., Moustaid-Moussa N. (2017). Regulation and Functions of the Renin-Angiotensin System in White and Brown Adipose Tissue. Compr. Physiol..

[275] Goh G.B., Pagadala M.R., Dasarathy J., Unalp-Arida A., Sargent R., Hawkins C., Sourianarayanane A., Khiyami A., Yerian L., Pai R. (2015). Renin-angiotensin system and fibrosis in non-alcoholic fatty liver disease. Liver Int..

[276] de Vries A.P., Ruggenenti P., Ruan X.Z., Praga M., Cruzado J.M., Bajema I.M., D’Agati V.D., Lamb H.J., Pongrac Barlovic D., Hojs R. (2014). Fatty kidney: Emerging role of ectopic lipid in obesity-related renal disease. Lancet Diabetes Endocrinol..

[277] Walker R.E., Ford J.L., Boston R.C., Savinova O.V., Harris W.S., Green M.H., Shearer G.C. (2020). Trafficking of nonesterified fatty acids in insulin resistance and relationship to dysglycemia. Am. J. Physiol. Endocrinol. Metab..

[278] Pawlak M., Lefebvre P., Staels B. (2015). Molecular mechanism of PPARalpha action and its impact on lipid metabolism, inflammation and fibrosis in non-alcoholic fatty liver disease. J. Hepatol..

[279] Targher G., Byrne C.D. (2017). Non-alcoholic fatty liver disease: An emerging driving force in chronic kidney disease. Nat. Rev. Nephrol..

[280] Verweij N., Haas M.E., Nielsen J.B., Sosina O.A., Kim M., Akbari P., De T., Hindy G., Bovijn J., Persaud T. (2022). Germline Mutations in CIDEB and Protection against Liver Disease. N. Engl. J. Med..

[281] Abul-Husn N.S., Cheng X., Li A.H., Xin Y., Schurmann C., Stevis P., Liu Y., Kozlitina J., Stender S., Wood G.C. (2018). A Protein-Truncating HSD17B13 Variant and Protection from Chronic Liver Disease. N. Engl. J. Med..

